# TAK-676: A Novel Stimulator of Interferon Genes (STING) Agonist Promoting Durable IFN-dependent Antitumor Immunity in Preclinical Studies

**DOI:** 10.1158/2767-9764.CRC-21-0161

**Published:** 2022-06-23

**Authors:** Elizabeth Carideo Cunniff, Yosuke Sato, Doanh Mai, Vicky A. Appleman, Shinji Iwasaki, Vihren Kolev, Atsushi Matsuda, Judy Shi, Michiyo Mochizuki, Masato Yoshikawa, Jian Huang, Luhua Shen, Satyajeet Haridas, Vaishali Shinde, Chris Gemski, Emily R. Roberts, Omid Ghasemi, Hojjat Bazzazi, Saurabh Menon, Tary Traore, Pu Shi, Tennille D. Thelen, Joseph Conlon, Adnan O. Abu-Yousif, Christopher Arendt, Michael H. Shaw, Masanori Okaniwa

**Affiliations:** 1 Takeda Development Center Americas, Inc. (TDCA), Lexington, Massachusetts.; 2 Takeda Pharmaceutical Company, Ltd., Fujisawa, Kanagawa, Japan.

## Abstract

Oncology therapies targeting the immune system have improved patient outcomes across a wide range of tumor types, but resistance due to an inadequate T-cell response in a suppressive tumor microenvironment (TME) remains a significant problem. New therapies that activate an innate immune response and relieve this suppression may be beneficial to overcome this hurdle. TAK-676 is a synthetic novel stimulator of interferon genes (STING) agonist designed for intravenous administration. Here we demonstrate that TAK-676 dose-dependently triggers activation of the STING signaling pathway and activation of type I interferons. Furthermore, we show that TAK-676 is a highly potent modulator of both the innate and adaptive immune system and that it promotes the activation of dendritic cells, natural killer cells, and T cells in preclinical models. In syngeneic murine tumor models *in vivo,* TAK-676 induces dose-dependent cytokine responses and increases the activation and proliferation of immune cells within the TME and tumor-associated lymphoid tissue. We also demonstrate that TAK-676 dosing results in significant STING-dependent antitumor activity, including complete regressions and durable memory T-cell immunity. We show that TAK-676 is well tolerated, exhibits dose-proportional pharmacokinetics in plasma, and exhibits higher exposure in tumor. The intravenous administration of TAK-676 provides potential treatment benefit in a broad range of tumor types. Further study of TAK-676 in first-in-human phase I trials is ongoing.

**Significance:**

TAK-676 is a novel systemic STING agonist demonstrating robust activation of innate and adaptive immune activity resulting in durable antitumor responses within multiple syngeneic tumor models. Clinical investigation of TAK-676 is ongoing.

## Introduction

Immuno-oncology checkpoint inhibitor therapies are revolutionizing cancer treatment and have improved patient outcomes across a broad range of tumor types; however, only a small fraction of patients respond to therapy, and primary and secondary resistance remains a significant problem (1–3). Nonresponsiveness to immunotherapy may involve an inadequate T-cell response in the tumor microenvironment (TME; ref. 1). Thus, there is an urgent need to identify new therapies that activate both the innate and adaptive immune response to drive more potent and durable antitumor immunity, with a manageable safety and tolerability profile.

Type I IFNs are known for promoting robust antiviral immunity. Accumulating evidence suggest that type I IFNs produced by innate immune cells, such as dendritic cells (DCs), play a critical role in cancer immunosurveillance. Type I IFN production has been associated with the host detection of danger signals that are released from stressed, injured, or necrotic cells through a diverse family of innate pattern recognition sensors (1). One such sensor is the stimulator of interferon genes (STING). STING is an endoplasmic reticulum signaling protein, that is broadly expressed in both immune and nonimmune cell types. STING binds 2′,3′-cGAMP produced by cyclic GMP–adenosine monophosphate (AMP) synthase in response to cytosolic DNA (4) and rapidly induces type I IFNs and proinflammatory cytokines (5–7). It has been shown that productive type I IFN-mediated antitumor immunity requires an intact STING pathway within the DC population (8). Collective evidence highlights that agonism of the host STING pathway plays a critical role in eliciting robust and durable antitumor immunity (9, 10).

Recognizing the therapeutic potential of targeting the STING pathway for its positive immune-modulatory properties has prompted many groups to develop STING-activating drugs utilizing intratumoral injection as an administration route (9). Poor pharmacokinetic and physicochemical properties of 2′,3′-cGAMP and its derivatives are hurdles to the development of systemically administered STING agonists (9) . Therefore, overcoming these obstacles would facilitate the clinical development of STING agonists as novel anticancer therapeutics and potentially increase the number of patients that can benefit. TAK-676 is a novel synthetic STING agonist that is administered as an intravenous infusion and is a highly potent modulator of the innate immune system (11, 12). TAK-676 is currently under investigation in cancer patients with locally advanced or metastatic solid tumors (ref. 13; NCT04420884, NCT04879849, NCT04541108). These trials are investigating the antitumor effects of TAK-676 as a monotherapy or with combination therapies such as checkpoint inhibitors, radiation, and chemotherapy.

Here we present the first report of the structure of TAK-676, and the activity of TAK-676 in preclinical studies. We demonstrate that TAK-676 dose-dependently triggers activation of the STING signaling pathway and activation of type I IFNs, as well as robust activation of innate and adaptive immune activity both *in vitro* and *in vivo*. In syngeneic murine tumor models *in vivo,* TAK-676 induced dose-dependent cytokine responses and increased the activation and proliferation of immune cells within the TME and tumor-associated lymphoid tissue. Ultimately this immune activation resulted in durable antitumor responses within multiple syngeneic tumor models.

## Materials and Methods

A summary methodology for all components of the analyses reported is provided below; full methodologic details for all analyses are provided in the Supplementary Appendix. For details on cell lines and use, see “Cell lines and mouse models” section at the end of this Materials and Methods section.

### Chemical Synthesis and Characterization of TAK-676

Detailed methods for the synthesis and purification of TAK-676 can be found within the Supplementary Appendix.

### STING Binding and Pathway Activation

To measure the binding of TAK-676 to mouse, rat, monkey, and human STING orthologs, *in vitro* time-resolved fluorescence resonance energy transfer (TR-FRET) assays were used. To assess pathway activation, the stimulatory effect of TAK-676 on the STING signaling pathway *in vitro* in the THP1-Dual human acute myeloid leukemia (AML) and CT26.wild type (WT) and STING-deficient CT26.KO mouse colon carcinoma cell lines was evaluated. Cells were treated with increasing concentrations of TAK-676 for 3 hours, subsequently lysed, and the proteins analyzed by SDS-PAGE and immuno-blotting. For experiments in STING-deficient CT26.KO cell lines, 5,6-dimethylxanthenone-4-acetic acid (DMXAA; 50 μg/mL) was used as a control for a treatment time of 1 hour prior to cell lysis and blotting. Three STING-induced phosphorylation events, part of a signaling cascade critical for the induction of type I IFNs, were monitored by Western blotting: TANK-binding kinase 1 (TBK1) on S172 in the kinase activation loop, STING on S365/366 (mouse/human numbering), and interferon regulatory factor (IRF) 3 on S396.

STING agonists trigger the production of type I IFNs and the induction of interferon-stimulated genes (ISG) through IRFs. The potency of the STING agonist was evaluated in reporter assays using the ISRE_NanoLuc human embryonic kidney 293 (HEK293T) cells, the more physiologically relevant human monocyte derived THP1-Dual cells, and the mouse macrophage RAW-Lucia ISG cells. These cell lines, that are of mouse or human origin, allow measurement of STING agonist activity using the NanoLuc luciferase (HEK293T) or IRF-inducible Lucia luciferase as readouts (THP1-Dual and RAW-Lucia). In HEK293T cells, which lack endogenous STING, human and mouse STING isoforms were transiently transfected, and a reporter assay was developed by generating an ISRE_NanoLuc HEK293T stable cell line using the pNL (NLucP/ISRE/Hygro) vector to monitor the resulting increase in type I IFNs. The concentration at inflection point of the curve producing a half-maximal response (EC_50_) was calculated in cultured ISRE_NanoLuc HEK293T cells that were transiently transfected with human WT STING (R232), mouse STING (WT), and the four other isoforms of STING—R232H, R293Q, G230A-R293Q (AQ), and R71H-G230A-R293Q (HAQ)—that exist in human populations (14). The EC_50_ was also calculated in RAW-Lucia ISG cells and THP1-Dual cells. Digitonin was used to permeabilize the HEK293T and RAW-Lucia ISG cells, but not the THP1-Dual cell line as digitonin was toxic to these cells, thus preventing accurate measurement of type I IFNs.

### Generation of STING Knockout and WT Cell Line Clones in CT26.WT and B16F10 Cells

The “hit-and-run” clustered regularly interspaced short palindromic repeats (CRISPR) method was employed for creation of STING knockout (KO) cell clones and their WT counterparts in CT26.WT and B16F10 cells by following the Lipofectamine CRISPRMAX Transfection Reagent protocol (Thermo Fisher Scientific). Confluent clones were harvested and evaluated by Western blotting for mSTING protein and PCR analysis of mSTING genomic DNA to reconfirm efficient knockdown. Several confirmed clonal mSTING KOs, as well as matched WT controls shown to have intact mSTING, were expanded, banked, tested for murine pathogens, and cryopreserved for use in subsequent *in vitro* and *in vivo* studies.

### *In Vitro* Immune Cell Activation Assays

As STING proteins are highly expressed in DCs, the activation of DCs derived from human peripheral blood mononuclear cells from 5 healthy donors and mouse bone marrow (BMDC) was evaluated using flow cytometry to detect the expression of the DC activation marker cluster of differentiation (CD) 86. The human monocyte-derived DCs (MoDC) were differentiated from CD14^+^ cells (monocytes), and the mouse BMDCs were differentiated from freshly isolated bone marrow (BM) cells of femurs and tibiae of 2 donor BALB/c mice. Mean fluorescence intensity (MFI) of CD86^+^ DCs from each sample was evaluated by flow cytometry and MoDC viability (defined as LIVE/DEAD Fixable Near-IR Dead Cell Staining Dye negative population from the parent gate) was recorded. Natural killer (NK) and T-cell activation was assessed in whole blood from 5 healthy human donors using flow cytometry to detect expression of the activation marker CD69 on CD4^+^ and CD8^+^ T cells and CD56^+^/CD16^+^ NK cells. CD69 expression (as evaluated by MFI) was measured for each cell population, and mean plasma-based EC_50_, Hill slope, and concentration producing 10% of maximal response (EC_10_) were calculated.

### Assessment of Pharmacokinetics

Female BALB/c mice were inoculated subcutaneously with mouse A20 (B-cell lymphoma) tumor cells in the right flank and TAK-676 dosing was initiated when estimated tumor volumes reached 300–800 mm^3^. Mice (*n* = 3/group) were dosed via intravenous injection with single doses of TAK-676 at 0.025, 0.125, 0.25, 0.5, and 2 mg/kg formulated in PBS as a single agent. Exposure to TAK-676 was determined by measuring plasma and tumor concentrations of TAK-676 at various timepoints using a LC-MS/MS method.

### *In Vivo* Assessment of TAK-676 Efficacy in Mouse Models

TAK-676 activity was evaluated in syngeneic CT26.WT and A20 mouse models. In addition, a B16F10_WT (melanoma) tumor model and STING-deficient B16F10_STING KO tumor model were used to demonstrate the STING dependence of antitumor activity using both WT and STING-deficient C57BL/6J-Tmem173gt/J (Goldenticket; Jackson Laboratory) models. Mice were inoculated subcutaneously into the right flank with CT26.WT tumor cells, A20 tumor cells, or B16F10 tumor cells and were treated with either vehicle (PBS) or TAK-676. Mice in the vehicle and TAK-676 (1 or 2 mg/kg) groups received intravenous doses once every 3 days for three doses (Q3D×3, days 0, 3, and 6). Effects on tumor growth were evaluated by measuring growth rate inhibition (GRI) and tumor regression. Tolerability was assessed by measuring percent body weight loss (BWL; body weights were measured twice weekly), mortality, or any clinical signs of adverse treatment-related side effects (see Supplementary Appendix).

### Assessment of *In Vivo* Pharmacodynamic Effects

#### Cytokine Induction

Pharmacodynamic cytokine response to TAK-676 was evaluated in female BALB/c mice inoculated subcutaneously with 1.0 × 10^6^ A20 syngeneic tumor cells, and in C57BL/6 WT or Goldenticket mice inoculated subcutaneously with 0.8 × 10^6^ B16F10 WT or STING KO tumor cells. When tumor volumes grew to 300–800 mm^3^, mice (*n* = 5/group) were dosed once with vehicle (PBS), or TAK-676 intravenously at 0.05, 0.125, 0.5, 1.0, and 2.0 mg/kg. The pharmacodynamic effect of TAK-676 in plasma (for animals inoculated with A20 cells) or serum (for animals inoculated with B16F10 cells) and tumor samples was profiled to quantitate the following cytokines: IFNα, IFNγ, IFNγ-induced protein 10 (IP-10) for all mice; TNFα, IL6, and monocyte chemoattractant protein-1 (MCP-1) for animals inoculated with A20 cells.

#### *In Vivo* Immune Cell Activation and Proliferation

Female C57BL/6 mice bearing B16F10 tumors were treated with TAK-676 intravenously at 0.3, 1.0, 2.0 mg/kg or vehicle on day 0 (*n* = 4/group). In addition, female BALB/c mice bearing CT26 tumors were treated with vehicle or 0.25 mg/kg TAK-676 on day 0 (*n* = 4/group). Pooled tumor-draining lymph nodes (axillary, brachial, and inguinal) and tumors were collected on day 3, and immune cell populations were analyzed by flow cytometry. Samples were initially gated off total live cells (within the live/dead negative gate) and subsequently for CD45^+^ cells. In the tumor, total T cells, defined as CD3^+^, were then examined further to specifically identify tumor-infiltrating CD8^+^ T cells. Because of the strong correlation between the presence of cytotoxic CD8^+^ T cells within the tumor and clinical prognosis (15, 16), CD8^+^ T cells in the tumor samples were further characterized for markers of activation and proliferation. CD8^+^ T-cell populations were evaluated for either CD69 (A20 tumors) or Ki67 and CD25 expression (CT26 tumors). In addition, to assess the functionality of the CD8^+^ T-cell population within the A20 tumors, expression of the proinflammatory cytokine IFNγ was evaluated *ex vivo* following an overnight stimulation with plate bound α-CD3 and soluble α-CD28 antibodies. In the lymph node, CD8^+^ T cells were also assessed for IFNγ expression, as well as Ki-67 and CD69 expression, following overnight stimulation with α-CD3/αCD28. Lymph nodes were also evaluated for the CD11c^+^ MHC1^+^ DC population as a fraction of all viable CD45^+^ cells, as well as for the expression of DC activation markers CD80 and CD86. Lymph nodes from mice inoculated with CT26 tumors were also evaluated for the expression of CD69 on NK cells.

### Cell Lines and Mouse Models

All animal experiments were performed in compliance with protocols as approved by an Institutional Animal Care and Use Committee. All cell lines used in the experiments were obtained from the indicated sources summarized below and included in Supplementary Table S1, cultured in appropriate media and expanded, and then banked into the Takeda Oncology Cell bank as high-density frozen vials stored in liquid nitrogen (LN_2_) between passage three and six. For cell line reporter assays, cells were thawed from high-density frozen vials and then used immediately for experiments following a short recovery period. Cells for *in vitro* Western blot assays and for *in vivo* implantation were thawed from frozen vials and cultured a short time before use in the experiment. During culture time, cells were fed and split as needed to avoid over confluence. Cells were used for experiments as needed during the period of culture but never exceeding passage 10. Cells were *Mycoplasma* and murine pathogen tested via the IMPACT I assay by Idexx Bioresearch (the most recent testing dates are indicated in Supplementary Table S1 and summarized below). CT26.WT, A20, and B16F10 cell lines were additionally verified using IDEXX Cell Check Cell Line Authentication Service with 27-Marker STR Strain Analysis.

Individual cell line details: THP-1 Dual Huamn Acute Myeloid Leukemia cells were obtained from Invivogen (catalog no. thpd-nfis) and were last *Mycoplasma* tested on June 19, 2017. Human Embryonic Kidnsey 293 (HEK293T) cells were obtained from ATCC (catalog no. CRL-11268) and were last *Mycoplasma* tested on February 15, 2018. ISRE-Nano Luc HEK293T cells were generated by Promega with Vector CS190901 and were last *Mycoplasma* tested on February 15, 2018. CT26.WT cells were obtained from ATCC (catalog no. CRL-2638) and were last *Mycoplasma* tested on June 23, 2016. A20 cells were obtained from ATCC (catalog no. TIB-208) and were last *Mycoplasma* tested on June 25, 2015. B16F10 cells were obtained from ATCC (catalog no. CRL-6475) and were last *Mycoplasma* tested on July 22, 2016.

### Statistical Analysis

For *in vivo* studies, the differences in the tumor growth trends over time between pairs of treatment groups were assessed by fitting each animal's data to a simple exponential growth model and comparing the mean growth rates of the two groups. The difference in the growth rates was summarized by the GRI, which is the reduction in growth rate experienced by the treatment group relative to that of the reference group, expressed as a fraction of the vehicle growth rate. A positive GRI indicates that the tumors in the treatment group grew at a reduced rate relative to the reference group. A statistically significant *P* value (<0.05) suggests that the trends over time for the two treatment groups were different. For all other relevant studies, the mean, SD, and *P* value (where significant; relative to control) were calculated using GraphPad Prism 7 software (GraphPad Software).

### Data Availability Statement

Data are available upon reasonable request made to the corresponding authors.

## Results

### TAK-676, a Synthetic STING Agonist, is a Potent and Selective Binder of STING Proteins of Multiple Species

**FIGURE 1:**
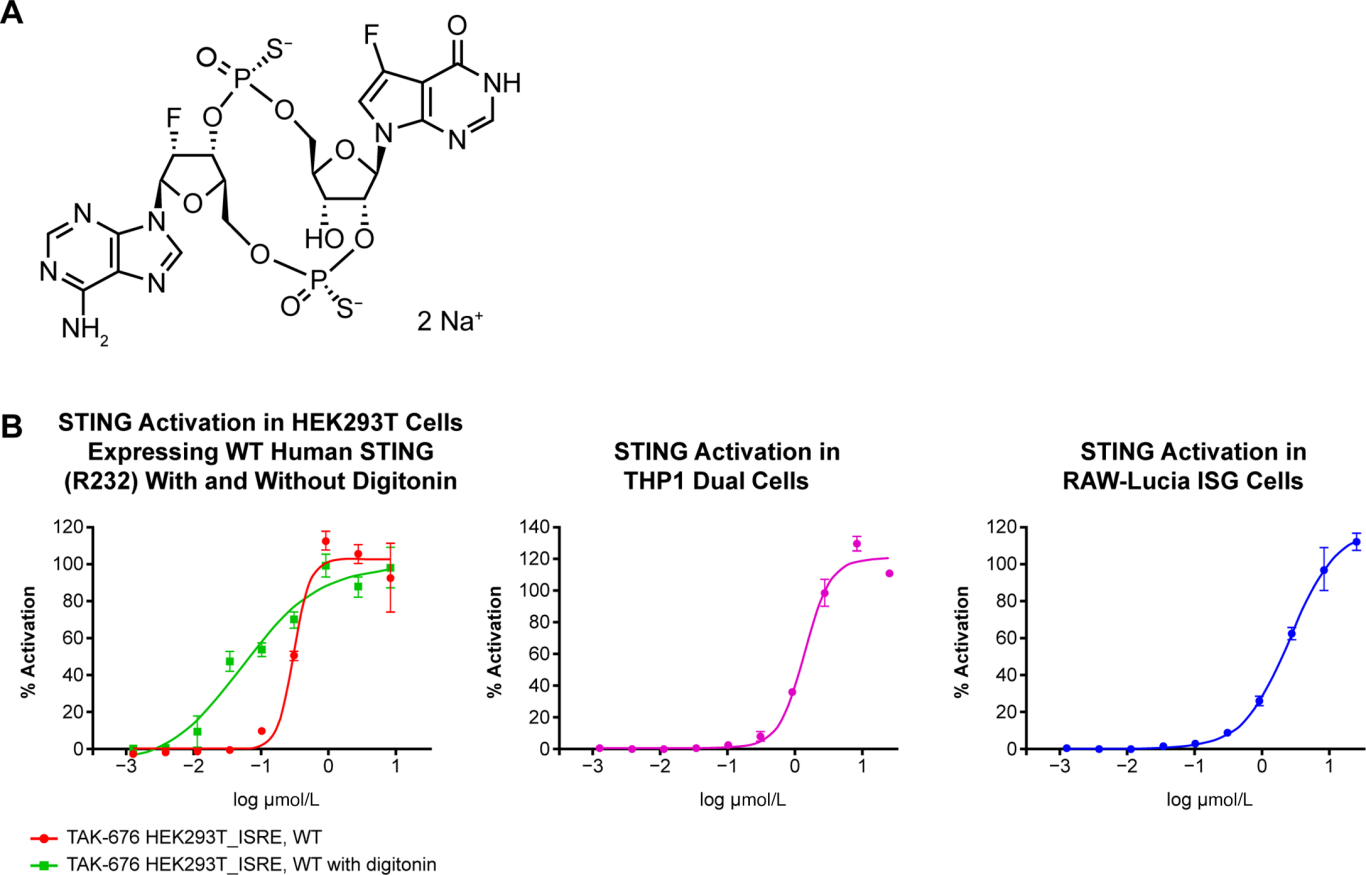
Structure of STING agonist TAK-676 (**A**) and *in vitro* STING activation by TAK-676 in HEK293T, THP1-Dual and RAW-Lucia ISG cells (**B**). All data shown are representative of at least three independent experiments. WT, wild type.

The chemical structure of TAK-676 is shown in Fig. 1A (for details, see Supplementary Appendix: Methods of chemical synthesis and characterization of TAK-676). Data on TAK-676 binding from the TR-FRET assay showed highly selective binding and activation of STING proteins from various species, with dissociation constant (K_d_) values estimated to be less than or equal to 0.010 ± 0.0008 μmol/L, 0.008 ± 0.001 μmol/L, 0.011 ± 0.001 μmol/L, and 0.027 ± 0.008 μmol/L for mouse (*n* = 3), rat (*n* = 3), cynomolgus monkey (*n* = 3), and human (*n* = 20) STING orthologs, respectively (Supplementary Table S2).

### TAK-676 Dose-Dependently Triggers Activation of the STING Signaling Pathway and Type I IFNs

IFN activation was assessed *in vitro* by using reporter assays. ISRE_NanoLuc HEK293T cells were transiently transfected with human WT STING (R232), with and without digitonin. The EC_50_ was 0.3 ± 0.11 μmol/L in the absence of digitonin and 0.09 ± 0.07 μmol/L in permeabilized cells. IFN activation EC_50_ values were also assessed in commercially available THP1 Dual and RAW-Lucia ISG reporter cell lines and EC_50s_ were 1.53 ± 0.45 μmol/L, and 1.78 ± 0.48 μmol/L in the THP1-Dual, and RAW-Lucia ISG cell lines, respectively (Fig. 1B).

**FIGURE 2:**
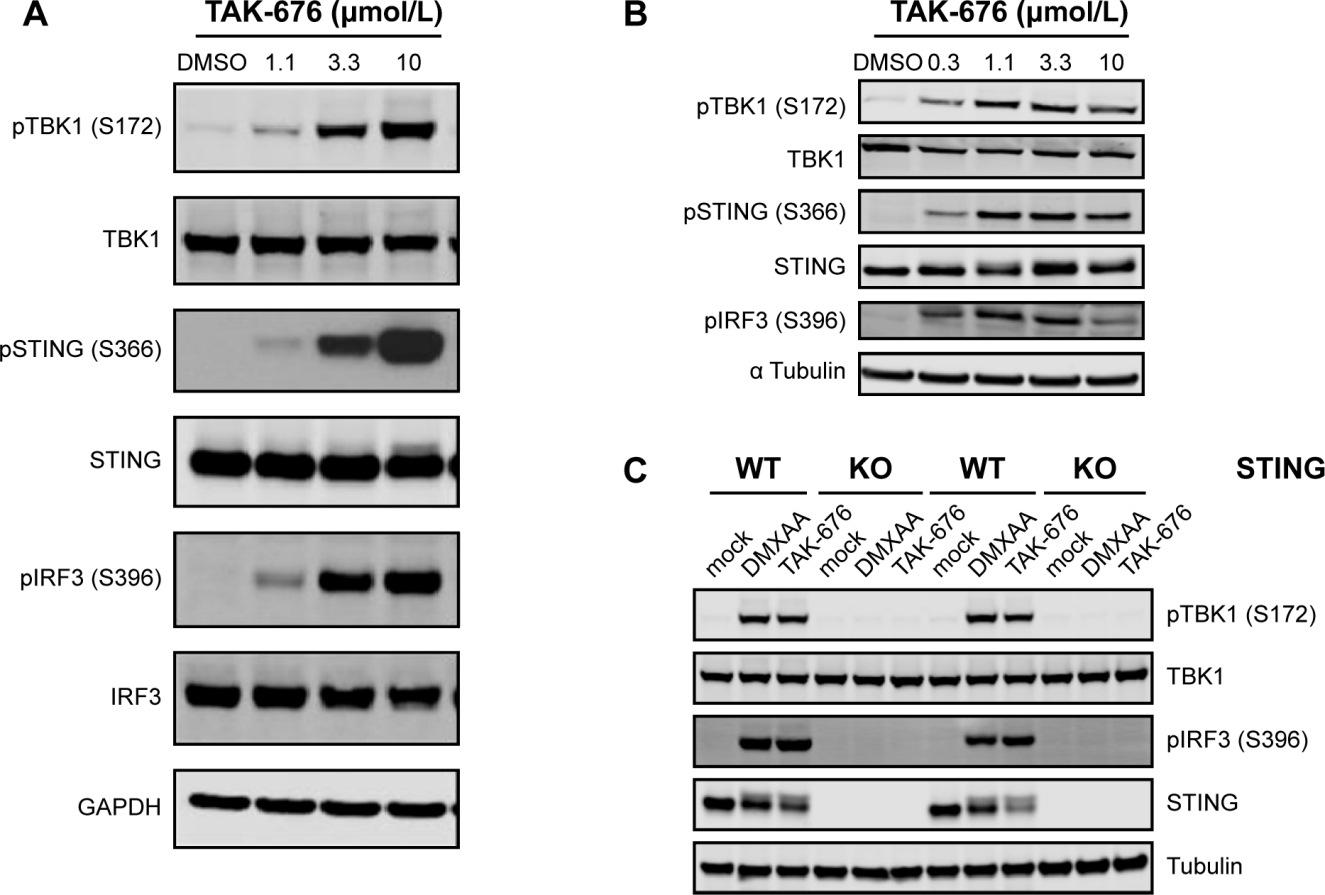
TAK-676 dose-dependently activates the STING-TBK1-IRF3 pathway in THP1-Dual human AML cells (**A**) and CT26.WT cells (**B**). This activation is lost in STING-deficient CT26.WT cells following treatment with DMXAA (50 μg/mL, 1 hour) or TAK-676 (6.6 μmol/L, 2 hours) in **C**. Results are shown for two distinct STING KO clones and two distinct WT clones. All data shown are representative of at least three independent experiments. DMSO, dimethyl sulfoxide; GAPDH, glyceraldehyde 3-phosphate dehydrogenase; p, phosphorylated.

To assess the ability of TAK-676 to activate the STING pathway, the phosphorylation of STING and STING pathway proteins was evaluated in human THP1-Dual and murine CT26.WT cell lines following treatment with TAK-676, as shown in Fig. 2A and B, respectively. These data demonstrate a dose-dependent induction in the expression of pSTING (S366), pTBK1 (S172), and pIRF3 (S396) in both murine and human cell lines following treatment with TAK-676. To confirm that the induction of STING pathway activation following treatment with TAK-676, STING-deficient cells were generated from CT26.WT cell lines. As shown in Fig. 2C, in the absence of STING expression neither TAK-676 nor control STING agonist DMXAA induced the phosphorylation of pTBK1 (S172) or pIRF3 (S396). Collectively, these data demonstrate the ability of TAK-676 to dose-dependently induce STING-TBK1-IRF3 activation is critically dependent on STING expression.

### TAK-676–Mediated STING Pathway Agonism Activates DCs, NK, and T Cells *In Vitro*

**FIGURE 3:**
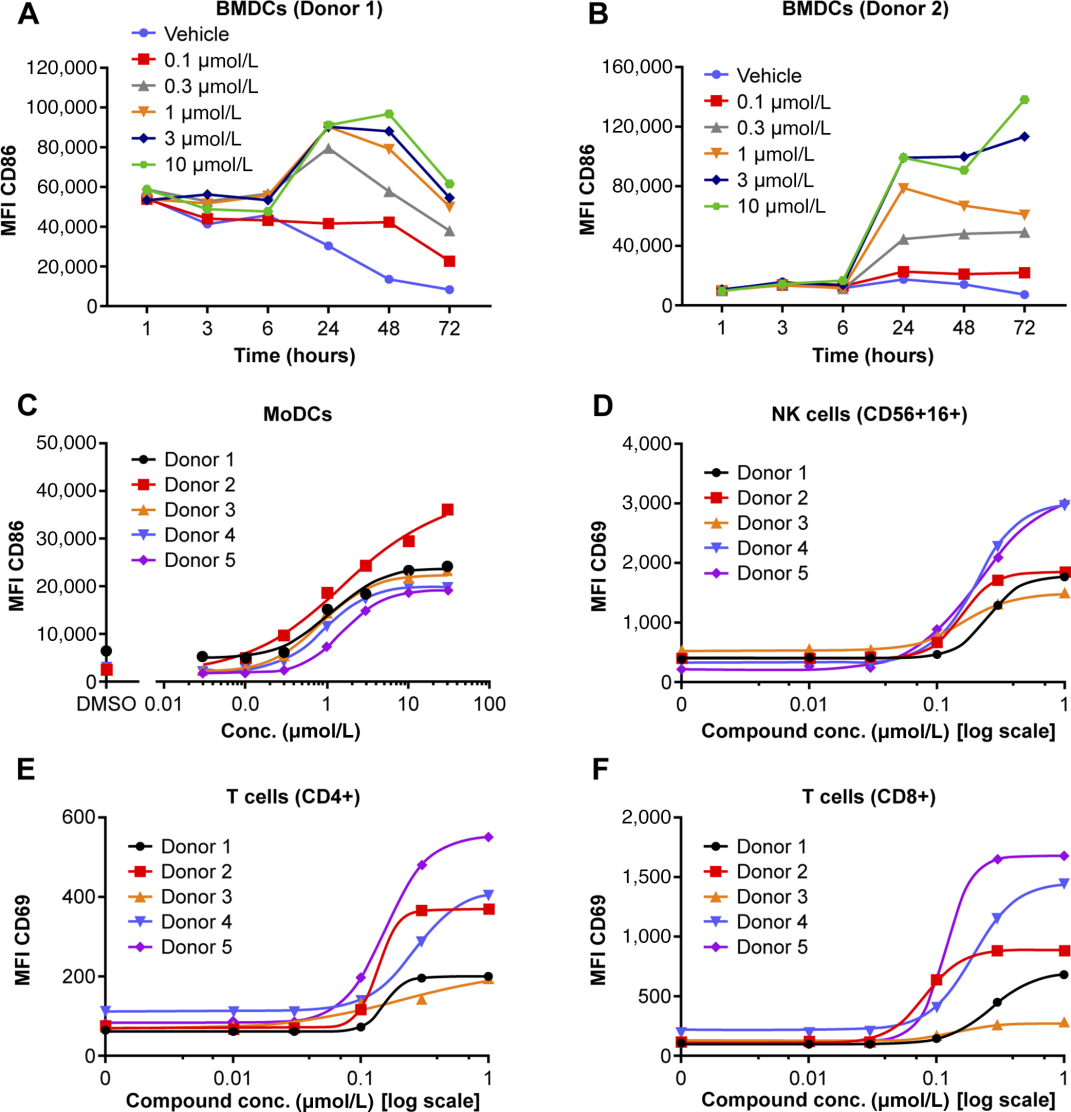
*In vitro* immune cell activation following treatment with TAK-676 in mouse BM-derived dendritic cells from 2 donors (**A** and **B**). **C,** Concentration-dependent activation in human MoDCs from 5 donors. NK-cell activation (**D**) and T-cell activation (**E** and **F**) in human whole blood from 5 donors following TAK-676 treatment for 24 hours. Conc., concentration; DMSO, dimethyl sulfoxide.

STING protein is highly expressed in DCs, and activation of DCs following STING pathway activation is key to promote innate and adaptive immune response (17). TAK-676–mediated DC activation studies were completed by monitoring the induction of the DC maturation marker CD86 by flow cytometry in both mouse BMDCs and human MoDCs. Fig. 3A–C demonstrate a dose-dependent TAK-676 induction of CD86 on the surface of BMDCs (0.1–10 μmol/L, 2 donors, 1, 3, 6, 24, 48, 72 hours) and MoDCs (0.03–30 μmol/L, 5 donors, 24 hours). The average EC_50_ value for TAK-676-induced MoDC activation at the 24-hour timepoint was 1.217 μmol/L (±0.352 μmol/L), and 0.32 μmol/L for the BMDC activation. No significant decrease of cell viability was observed in either MoDCs or BMDCs (Supplementary Fig. S1).

To further characterize the ability of TAK-676 to mediate both innate and adaptive immune cell activation, TAK-676–mediated NK- and T-cell activation was measured by evaluating the expression levels of CD69 on human peripheral blood NK and T cells (*n* = 5 healthy human donors), as shown in Fig. 3D–F. TAK-676 treatment resulted in a dose-dependent increase in CD69 expression in both NK and T cells. The plasma-based EC_50_ and Hill slope for each donor was analyzed, and the mean EC_50_ was estimated to be 0.271, 0.216, and 0.249 μmol/L for NK, CD8^+^, and CD4^+^ T cells, respectively. The mean plasma-based EC_10_ was calculated from the mean plasma-based EC_50_ to be 0.114, 0.0986, and 0.106 μmol/L for NK cells, CD8^+^, and CD4^+^ T cells, respectively. Collectively, these data suggest that TAK-676 is a potent inducer of both innate and adaptive immune cell activation.

### TAK-676 Exhibits Dose-Proportional Pharmacokinetics in Plasma and Greater Exposure in Tumor Tissue

To support systemic dosing of TAK-676, mean plasma and tumor concentration–time curves of TAK-676 in female BALB/c mice bearing A20 syngeneic tumors were calculated and are presented in Supplementary Fig. S2. As shown, intravenous administration of TAK-676 resulted in dose-proportional pharmacokinetics in the plasma and pharmacologically meaningful exposure in the tumor tissue versus plasma. The mean maximum observed concentration (*C*_max_) and mean area under the concentration–time curve (AUC) from time 0 to 72 hours (AUC_72_) after intravenous administration of TAK-676 0.025, 0.125, 0.25, 0.5, and 2 mg/kg are shown in Supplementary Table S3.

### TAK-676 Dosing Results in Significant T Cell–Dependent *In Vivo* Antitumor Activity

**FIGURE 4:**
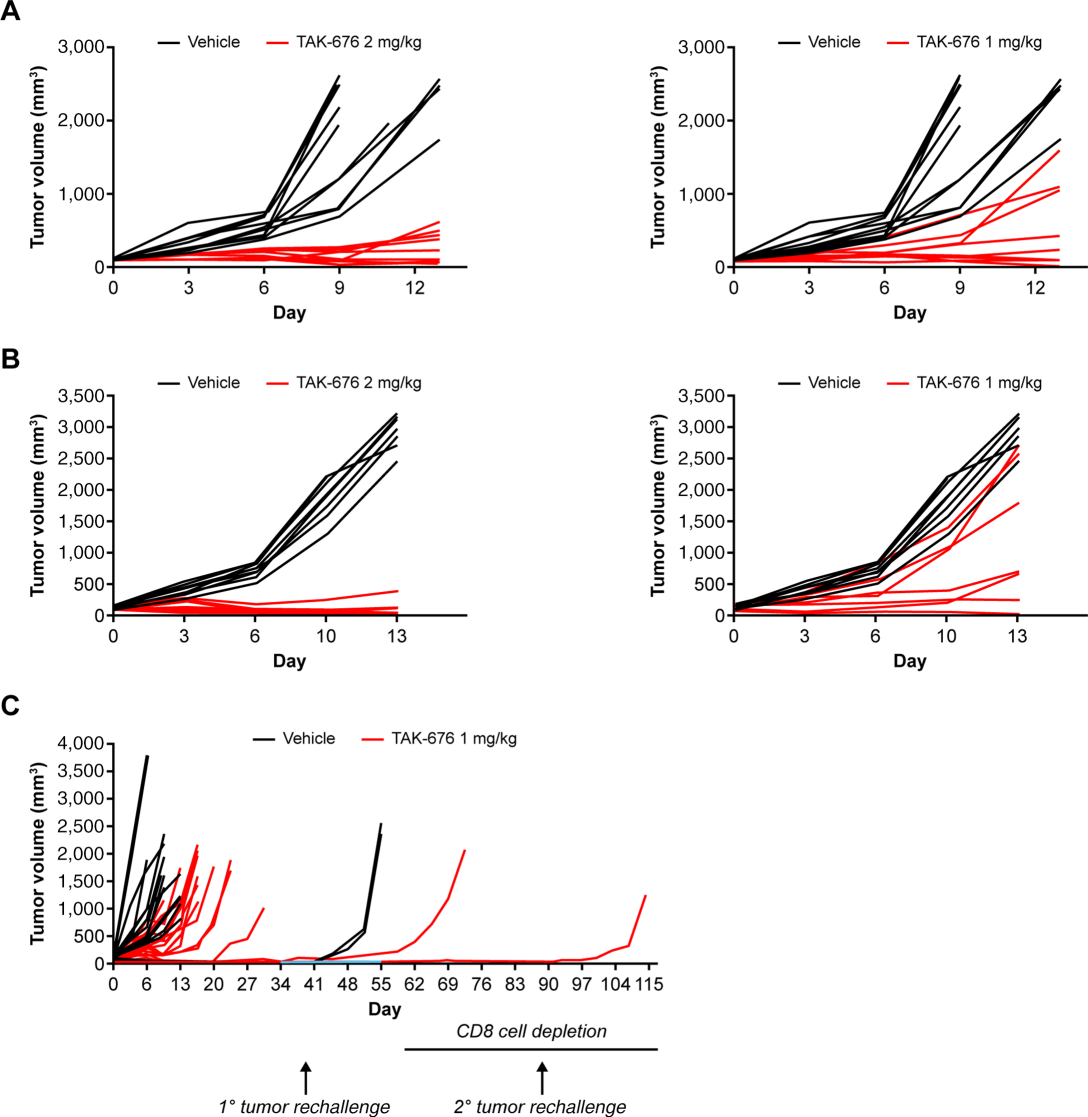
Antitumor effect of TAK-676 in: **A,** BALB/c mice bearing A20 syngeneic tumors; **B,** BALB/c mice bearing CT26.WT syngeneic tumors; **C,** BALB/c mice bearing CT26.WT syngeneic tumors following CR and subsequent rechallenge. Blue line denotes the period of rechallenge without CD8^+^ T-cell depletion. All data shown are representative of at least three independent experiments.

Dose-dependent *in vivo* efficacy of TAK-676 was tested in multiple mouse syngeneic tumor models, including A20 (Fig. 4A) and CT26.WT (Fig. 4B) syngeneic tumor models. In both models, the dosing of TAK-676 at both 1.0 and 2.0 mg/kg was well tolerated (Supplementary Fig. S3A and S3B). In mice, body weight is a meaningful safety readout to determine the MTD (18, 19), and all experiments were conducted at or below the MTD. BALB/c mice bearing A20 syngeneic tumors showed significant antitumor activity compared with vehicle treatment when TAK-676 was dosed at 1 mg/kg intravenously Q3D×3 (GRI 72%, *P* < 0.001), with 1 of 10 mice achieving complete response (CR). Similar to the A20 tumor model, CT26 tumor-bearing animals treated at 1 mg/kg also displayed tumor control over vehicle treated animals (Fig. 4B). Notably, when the dose of TAK-676 was increased from 1.0 to 2.0 mg/kg, there was an observable increase in the antitumor activity in both A20 (GRI = 91%, *P* < 0.001) and CT26 (GRI = 132%, *P* < 0.001) models (Fig. 4A and B; left), with more animals achieving CRs (two for A20 and four CRs for CT26). To assess the durability of the antitumor response elicited by TAK-676, mice with CRs in both groups were continuously monitored for ≥48 days with no tumor regrowth.

The ability of TAK-676 to promote durable CD8-dependent antitumor efficacy was assessed in a subsequent study in CT26.WT tumor-bearing BALB/c mice. In this study, two mice with CRs following treatment with 1 mg/kg of TAK-676 were rechallenged on the opposite flank with CT26.WT tumors on day 34. Following rechallenge, no tumor regrowth was observed in the complete responder mice, as compared with naïve control animals. To determine whether CD8^+^ T cells were responsible for the observed durable antitumor immunity in these two animals, on day 59, CD8^+^ T cells were depleted by antibody treatment, and regrowth was observed in one animal. At day 88, CD8^+^ T cells were depleted a second time, at which point regrowth was observed in the second animal (Fig. 4C). These data demonstrate that TAK-676 drives durable anti-tumor efficacy that is resistant to rechallenge and is dependent upon CD8^+^ T cells.

### TAK-676’s *In Vivo* Antitumor Activity is Dependent on STING Expression in Immune Cells

**FIGURE 5:**
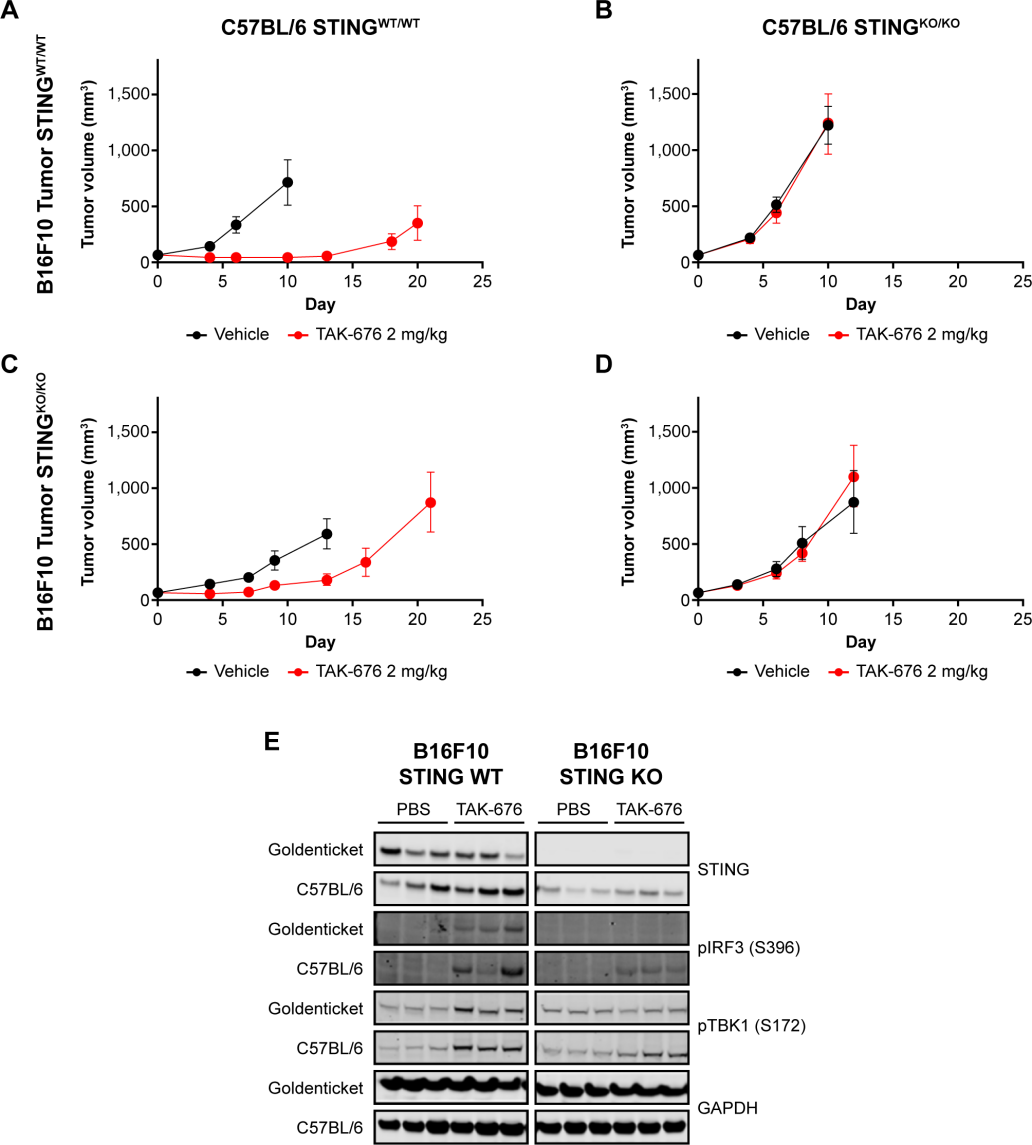
**A–D,** Mean tumor volume over time in WT (**A** and **C**) or STING-deficient C57BL/6J-Tmem173gt/J (Goldenticket; **B** and **D**) mice bearing STING WT (**A** and **B**) or deficient B16F10 (**C** and **D**) syngeneic tumors dosed with vehicle or TAK-676, and STING pathway activation (**E**) in STING WT or deficient B16F10 tumors implanted in WT C57BL/6 mice or STING-deficient Goldenticket mice.

To assess whether TAK-676–driven antitumor response was dependent on either host or tumor cell STING expression, Goldenticket mice were utilized and compared with WT mice following implantation of either WT or STING KO B16F10 tumor cells. When TAK-676 was dosed at 2 mg/kg intravenously Q3D×3 in WT C57BL/6 mice bearing B16F10 WT tumors, significant antitumor activity was observed (GRI 136%, *P* < 0.001; Fig. 5A). In contrast, in STING-deficient mice bearing B16F10 WT tumors, TAK-676 treatment failed to induce significant antitumor activity, with a GRI of −2% (*P* = 0.862; Fig. 5B). In addition, although TAK-676 was well tolerated in both strains, STING-deficient animals treated with TAK-676 showed reduced BWL when compared with their WT counterparts (see Supplementary Results and Supplementary Fig. S4 for details of tolerability). These results underscore the importance of the STING signaling pathway in the host for TAK-676–driven efficacy. To determine the role of STING expression in tumor cells, STING KO B16F10 cells were implanted in WT C57BL/6 animals. Treatment with TAK-676 2 mg/kg intravenously Q3D×3 in this model resulted in reduced, but significant, antitumor activity (GRI 53%, *P* = 0.001). These data demonstrate that STING expression in the host is the major determinant in promoting robust antitumor immunity (Fig. 5C). In further support of this, treatment of STING KO B16F10 tumor-bearing STING-deficient mice with TAK-676 also resulted in a complete lack of antitumor activity similar to what was observed with WT B16F10 tumor-bearing Goldenticket mice (GRI −16%, *P* = 0.349; Fig. 5D).

To evaluate the role of host versus tumor cell STING expression on STING pathway activation, tumors from these experiments were assessed for expression of STING, pIRF3 (S396), and pTBK1 (S172) at 3 hours after treatment with TAK-676 2 mg/kg intravenously As shown in Fig. 5E (left), TAK-676 treatment induced expression of pIRF3 (S396) and pTBK1 (S172) in both WT and STING-deficient mice implanted with B16F10 WT tumors. In animals implanted with STING KO B16F10 tumors (Fig. 5E; right), activation of the STING pathway can be observed, albeit at a much reduced level in WT C57BL/6 mice, but is completely abrogated in STING-deficient mice.

### TAK-676 Induces Dose-Dependent Cytokine Responses *In Vivo*

**FIGURE 6:**
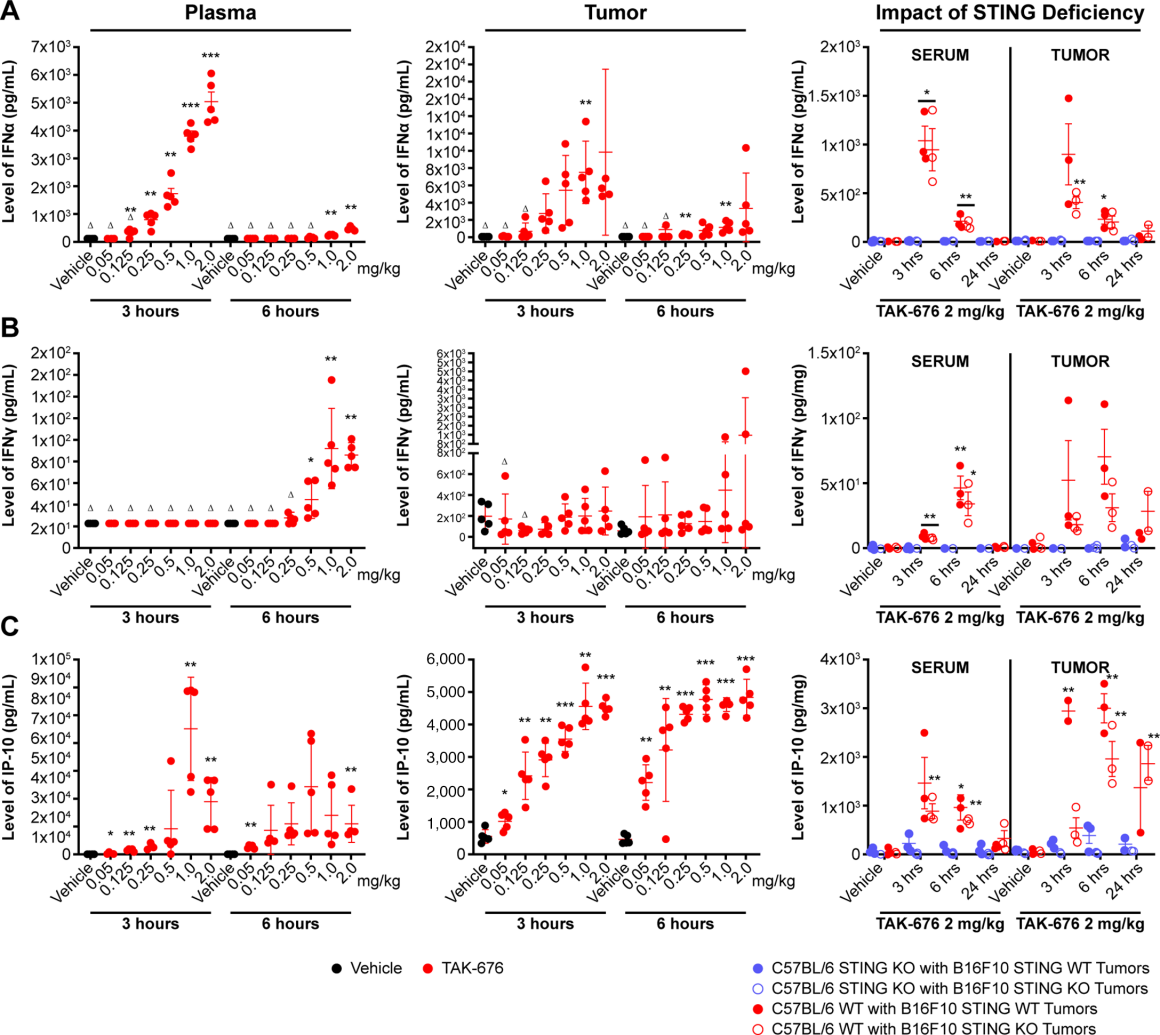
Cytokine responses in the plasma and tumor of A20 tumor-bearing mice (left, center, all data shown are representative of at least three independent experiments) and the impact of STING deficiency on those responses in B16F10 tumor-bearing mice (right) following exposure to a single intravenous dose of vehicle or TAK-676: IFNα (**A**); IFNγ (**B**); and IP-10 (**C**). Δ Indicates that some samples in this group had values below the lower limit of quantitation of the assay or had values extrapolated beyond the standard range. *, *P* ≤ 0.05 relative to vehicle control; **, *P* ≤ 0.01 relative to vehicle control; ***, *P* < 0.00001 relative to vehicle control; IP-10, IFNγ-induced protein 10.

Type I IFNs modulate the activity of innate immune effector cells and promote DC maturation and antigen presentation to T cells, propagating an adaptive immune response (20, 21) downstream of STING pathway activation. As such, we sought to investigate the potential of TAK-676 to induce type I IFNs, as well as other cytokine expression in the plasma and the tumor, following treatment in syngeneic tumor-bearing mice. As shown in Fig. 6A–C (IFNα, IFNγ, and IP-10) and Supplementary Fig. S5 (TNFα, MCP-1, and IL6), TAK-676 induced dose-dependent cytokine responses in female BALB/c mice bearing A20 syngeneic tumors. To determine whether the induction of these cytokines depended on either tumor or immune cell STING expression, cytokine responses were evaluated in the serum and tumors from both WT and STING-deficient mice bearing WT or STING-deficient B16F10 tumors following treatment with TAK-676. As shown in Fig. 6 (right), TAK-676–driven induction of IFNα, IFNγ, and IP-10 in both serum and tumor is dependent upon host STING expression and less so in the tumor cells. Collectively, the *in vivo* and *in vitro* data suggest that host STING expression is a critical determinant of robust immune activation leading to effective antitumor response.

### TAK-676 Increases the Activation and Proliferation of Immune Cells within the Tumor Microenvironment and Local Tumor-Associated Lymphoid Tissue

**FIGURE 7:**
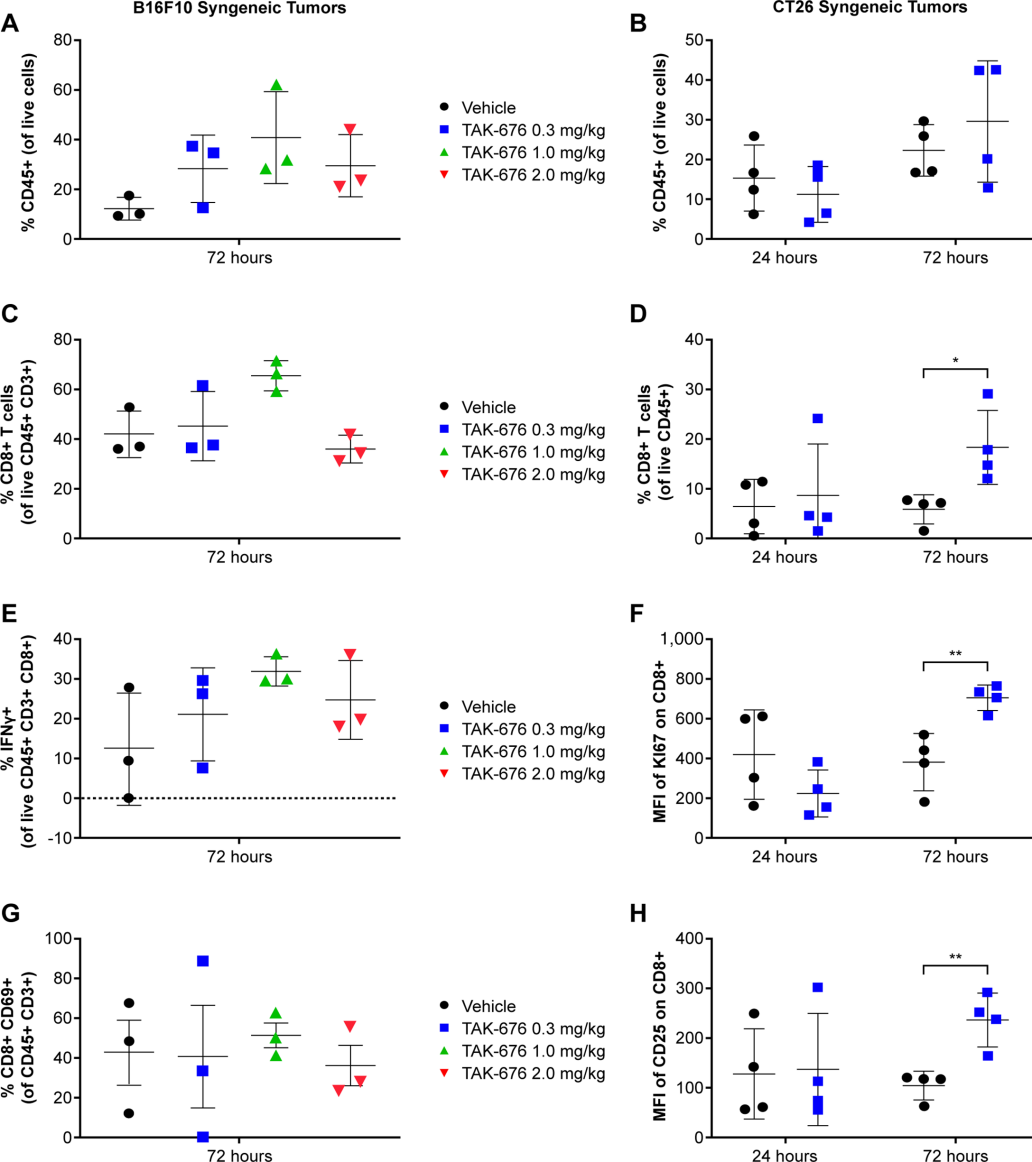
Effects of TAK-676 on immune cell populations within the tumor microenvironment in C57BL/6 mice bearing B16F10 syngeneic tumors (left) and BALB/c mice bearing CT26 syngeneic tumors (right). All data shown are representative of at least three independent experiments. **A,** Frequency of CD45^+^ cells in B16F10 tumors. **B,** Frequency of CD45^+^ cells in CT26 tumors. **C,** Frequency of CD8^+^ T cells in B16F10 tumors. **D,** Frequency of CD8^+^ T cells in CT26 tumors. **E,** Frequency of IFNγ^+^ CD8^+^ T cells in B16F10 tumors. **F,** MFI of Ki67 on CD8^+^ T cells in CT26 tumors. **G,** Frequency of CD8^+^ CD69^+^ T cells in B16F10 tumors. **H,** MFI of CD25 on CD8^+^ T cells in CT26 tumors. *, *P* ≤ 0.05; **, *P* ≤ 0.01. MHC, major histocompatibility complex.

To evaluate the ability of TAK-676 to induce T-cell infiltration, proliferation, and activation *in vivo*, immune modulation in the tumor and lymph nodes was assessed following treatment in tumor-bearing mice. The *in vivo* T-cell responses to TAK-676 in C57BL/6 mice bearing B16F10 tumors and BALB/c mice bearing CT26 tumors are shown in Fig. 7 (left and right, respectively). At day 3, the frequency of live CD45^+^ cells increased in tumors isolated from all TAK-676–treated groups (Fig. 7A and B). Intratumoral CD8^+^ T cells within the viable CD45^+^CD3^+^ cells were slightly increased at day 3 in B16F10 tumors, and significantly increased in CT26 tumors (Fig. 7C and D). While *in vitro* activation assays demonstrated an increase in CD69^+^ CD8^+^ T cells following treatment with TAK-676, this did not translate to *in* vivo assessments at day 3 in B16F10 tumors (Fig. 7G). However, an increase in IFNγ^+^CD8^+^ T cells was observed in B16F10 tumors, indicating activation of the CD8^+^ T cells (Fig. 7E). Corresponding increases in CD25 and Ki67 expression were also observed in the CD8^+^ T cells of CT26 tumors, further demonstrating CD8^+^ T-cell activation as well as increased proliferation in these tumors as early as day 3 following TAK-676 dosing (Fig. 7F and H).

**FIGURE 8:**
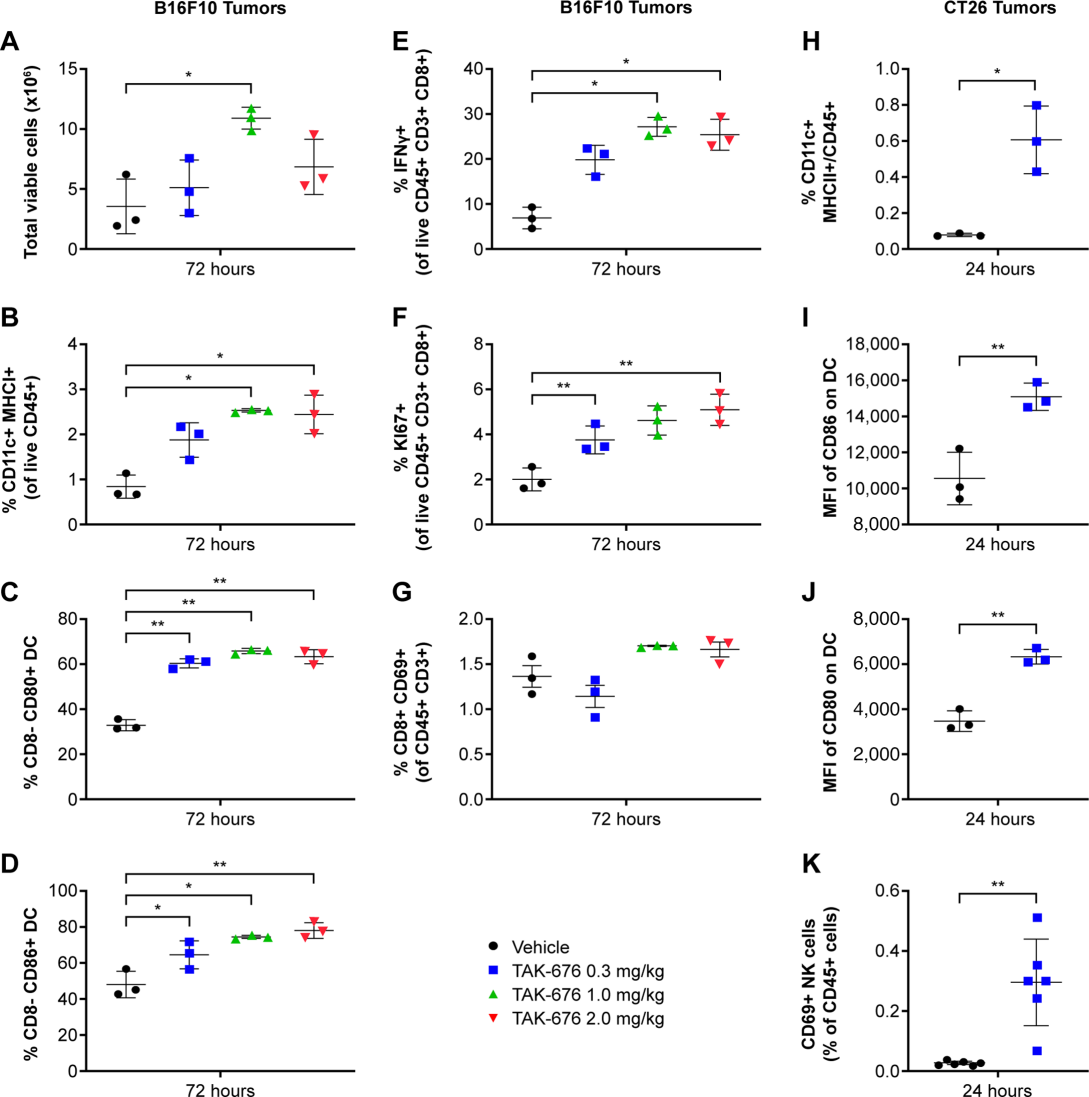
Effects of TAK-676 on immune cell populations within the tumor-draining lymph nodes from mice bearing B16F10 tumors (left and middle) and CT26 tumors (right). All data shown are representative of at least three independent experiments. **A,** Frequency of total viable cells. **B,** Frequency of CD11^+^ MHCI^+^ cells. **C,** Frequency of CD8^−^ CD80^+^ DCs. **D,** Frequency of CD8^−^ CD86^+^ DCs. **E,** Frequency of IFNγ^+^ CD8^+^ T cells. **F,** Frequency of Ki67^+^ CD8^+^ T cells. **G,** Frequency of CD69^+^ CD8^+^ T cells. **H,** Frequency of CD11c^+^ MHCII^+^ cells. **I,** MFI of CD86 on DCs. **J,** MFI of CD80 on DCs. **K,** Frequency of CD69^+^ NK cells. **, *P* ≤ 0.01.

Immune modulation following treatment with TAK-676 was also observed within the tumor-draining lymph nodes of B16F10 and CT26 tumor-bearing mice (Fig. 8). Within the tumor-draining lymph nodes of the B16F10 tumor-bearing mice, a measurable increase in total viable cells was initially observed at day 3 (Fig. 8A). Notably, also at day 3, the frequency of total DCs (identified as CD45^+^CD11c^+^MHCII^+^) within the lymph node was increased in both B16F10 and CT26 tumor-bearing mice (Fig. 8B and H). As observed in the *in vitro* DC activation assays, TAK-676 treatment also resulted in increased activation of DCs in the lymph nodes from both models at day 3, as demonstrated by increased frequency or expression of CD80 and CD86 (Fig. 8C, D, I, and J).

Similarly to what was observed within the tumors, an increase in CD69^+^ CD8^+^ T cells was not seen in the lymph nodes from B16F10 tumor-bearing animals (Fig. 8G). However, the frequency of IFNγ^+^CD8^+^ T cells was again increased within the tumor-draining lymph nodes of B16F10 tumor-bearing mice (Fig. 8E). In addition, TAK-676 induced the proliferation (as measured by Ki67^+^ staining) of CD8^+^ T cells within the lymph nodes of B16F10 tumor-bearing mice at day 3 following treatment (Fig. 8F). Finally, the frequency of CD69^+^ NK cells was increased in the lymph nodes from CT26 tumor-bearing animals, corresponding to the NK-cell activation observed *in vitro* (Fig. 8K).

Collectively, these data demonstrate increased NK-cell activation and DC frequency and activation in the lymph nodes accompanied by increased frequency, proliferation, and activation of CD8^+^ T cells in both the lymph nodes and the tumor following treatment with TAK-676. These data are consistent with the *in vitro* immune cell activation shown in Fig. 3, and confirm the ability of TAK-676 to induce both innate and adaptive immune responses *in vivo*.

## Discussion

This is the first report of the chemical structure and biological activity of the synthetic STING agonist TAK-676, designed for intravenous administration by improving the drug-like properties of natural STING agonists through chemical modifications. Here we demonstrate that TAK-676 is a potent and selective binder of STING proteins and activator of the STING signaling pathway with subsequent immune activation effects. Characterization of TAK-676 demonstrated potent activation of the STING signaling pathway in both human (THP1-Dual) and murine (CT26.WT) STING-expressing cell lines as evidenced by agonist-induced phosphorylation of TBK1, STING and the subsequent activation of IRF3, a key transcriptional regulator of type I IFN-dependent immune responses. TAK-676–mediated activation showed improved potency when cells were permeabilized with digitonin.

TAK-676 mediated STING pathway agonism and activated DCs, NK, and T cells both *in vitro* and *in vivo* in tumor-bearing syngeneic mouse models. The activation and priming of T cells observed here and with other STING agonists (22) to induce antitumor activity is of known importance in immunotherapy. The additional activation of NK cells seen here promotes an adaptive immune response by increasing cytokine and chemokine production, which enhances DC recruitment and maturation, and allows NK cells to directly kill tumors that T cells have not targeted (23, 24). The substantial activated immune cell response demonstrated with TAK-676 is important for driving the rejection of established tumors and distant metastases in addition to providing durable immunologic memory.

Significant STING-dependent antitumor activity was observed with TAK-676 compared with vehicle (PBS) in syngeneic mouse models *in vivo*. Mice bearing A20 tumor cells that experienced CRs in response to TAK-676 treatment did not show tumor regrowth over a 48-day period of monitoring, suggesting durable tumor suppression. In the Goldenticket mouse model TAK-676 treatment failed to induce significant antitumor activity against B16F10 syngeneic tumors, demonstrating that TAK-676 activity is mediated by STING agonism in the host, which is consistent with the presumed mechanism of action (11).

TAK-676 administered intravenously exhibited dose-proportional pharmacokinetics in plasma and pharmacologically meaningful exposure in the tumor tissue versus plasma. Direct intratumoral injection, which has been employed for clinical testing of some STING agonists (25), is designed to allow for drug penetration into the tumor lesion with lower concentrations of agent reaching healthy tissue (26). Furthermore, intratumoral administration requires needle accessible tumor lesions for treatment, therefore only some tumor types (melanoma, head and neck squamous cell carcinoma, hepatocellular carcinoma) have been clinically tested in humans (27), and there are legitimate concerns surrounding the risks of bleeding and tissue damage after repeated dosing (28). On the contrary, the intravenous administration with TAK-676 is potentially applicable to a broad population of patients with malignant cancers, including solid tumors and hematologic tumors, which may not be amenable to intratumoral administration. This expansion of tumor indications will afford opportunities to better understand how STING agonism modulates cancer immunity in humans.

Following administration in syngeneic tumor-bearing mice, TAK-676 dose-dependently induced cytokine production in both plasma and tumor, and increased the activation and proliferation of immune cells within the TME. Importantly, TAK-676 also exerted its effects within the tumor-draining lymph nodes, demonstrated by the increase in total viable cells, increased frequency of DCs, and increased activation and proliferation of CD8^+^ T cells over that of vehicle treated mice. DCs play a critical role in bridging an innate immune response into a long-term adaptive response with potential for durable memory (29, 30), and the ability of TAK-676 to increase the frequency of DCs within the tumor-draining lymph nodes is an important part of that process. The breadth of the immune response activated following TAK-676 administration suggests a broad-based effect that may be beneficial in terms of antitumor activity. Moreover, this response could be enhanced in combination with agents that disrupt DNA replication and increase antigen presentation, such as those used in radiation, chemotherapy, and anti-PD-1 therapy (31, 32).

In summary, TAK-676 is a selective and potent novel synthetic STING agonist that demonstrates immune activation effects in preclinical models bearing solid tumors as demonstrated by increased production of proinflammatory cytokines and activation of both tissue and circulating immune cells. Importantly, peripheral detection of TAK-676–mediated immune activation can be monitored in clinical samples, thereby enabling analysis of pharmacodynamic activity of TAK-676 in clinical studies (13). Significant STING-dependent antitumor activity was observed with no tumor regrowth and acceptable tolerability, suggesting durable tumor suppression. TAK-676 exhibited greater exposure in the tumor tissue than the plasma with intravenous administration, highlighting a potential benefit regarding the convenience of treating future patients versus intratumoral administration. Our findings identify TAK-676 as a promising new therapeutic candidate and support the clinical studies that are currently ongoing. TAK-676 is currently being investigated in phase I studies of patients with locally advanced or metastatic solid tumors alone or in combination with pembrolizumab, an anti-PD-1 antibody (ref. 13; NCT04879849, NCT04420884), and with various other anticancer agents (NCT04541108).

## Supplementary Material

Supplementary Methods SM1 Supplementary methods and results: The supplementary methods describe: 1) the chemical synthesis and characterization of TAK-676, inclusive of TAK-676 chemical structure, purification chromatogram and NMR characterization; 2) details regarding STING DNA cloning and purification (recombinant E. coli expression utilizing an N-terminal His tag and C-terminal Avi tag) for STING binding assays (time-resolved fluorescence resonance energy transfer assay); 3) description of pathway activation assays (inclusive of cell culture methods) in THP1-Dual™, CT26.WT, HEK293T (lack endogenous STING expression), and RAW-Lucia™ ISG cell lines; 4) methods describing the generation of STING knockout and WT cell line clones in CT26.WT and BF1610 cells via the CRISPR method; 5) details of immune cell activation assays in human dendritic cells, mouse dendritic cells, NK cells and T cells analyzed by flow cytometry; 6) details regarding inoculation of BALB/C mice with A20 cells (for generation of A20 syngeneic tumors) for pharmacokinetic analysis of TAK-676 administered intravenously; 7) details regarding inoculation of BALB/C (and Goldenticket mice with BF1610 tumor cells only) mice with CT26.WT, A20, and B16F10 tumor cells for in vivo antitumor activity analysis; 8) methods describing the in vivo pharmacodynamic effects of TAK-676 by cytokine induction and immune cell activation and proliferation. The supplementary results show that TAK-676 is tolerated in syngeneic mouse models supported by Supplementary Figure 3 (percentage body weight change in BALB/C mice [CT26.WT and A20 syngeneic tumors]) and Supplementary Figure 4 (percentage body weight change in STING-deficient Goldenticket mice [STING WT and B16F10 syngeneic tumors]).

Supplementary Figures S1-S5 Supplementary figures: Supplementary Figure 1 shows cell viability of human monocyte-derived dendric cells and mouse bone marrow dendritic cells. Supplementary Figure 2 shows mean plasma and tumor concentration-time curves of TAK-676 in BALB/C mice (A20 syngeneic tumors). Supplementary Figure 3 shows percentage body weight change in BALB/C mice (CT26.WT and A20 syngeneic tumors). Supplementary Figure 4 shows percentage body weight change in STING-deficient Goldenticket mice (STING WT and B16F10 syngeneic tumors). Supplementary Figure 5 shows cytokine expression in A20 tumor-bearing mice (plasma and tumor).

Supplementary Tables S1-S3 Supplementary tables: Supplementary Table 1 describes where cells were obtained from and details of authentication and mycoplasma testing dates. Supplementary Table 2 shows TAK-676 potency in cynomolgus monkey, human, mouse, and rat STING via time-resolved fluorescence resonance energy transfer assay. Supplementary Table 3 shows pharmacokinetic parameters (Cmax and AUC) after TAK-676 administration in BALB/C mice (A20 tumors).
