# Supplementary material for: TAK-676: A Novel Stimulator of Interferon Genes (STING) Agonist Promoting Durable IFN-dependent Antitumor Immunity in Preclinical Studies
Source: Cancer Res Commun. 2022 Jun 23;2(6):489–502. doi: 10.1158/2767-9764.CRC-21-0161 (PMC10010323; doi:10.1158/2767-9764.CRC-21-0161)
Supplement: Supplementary Methods SM1 — Supplementary methods and results: The supplementary methods describe: 1) the chemical synthesis and characterization of TAK-676, inclusive of TAK-676 chemical structure, purification chromatogram and NMR characterization; 2) details regarding STING DNA cloning and purification (recombinant E. coli expression utilizing an N-terminal His tag and C-terminal Avi tag) for STING binding assays (time-resolved fluorescence resonance energy transfer assay); 3) description of pathway activation assays (inclusive of cell culture methods) in THP1-Dual™, CT26.WT, HEK293T (lack endogenous STING expression), and RAW-Lucia™ ISG cell lines; 4) methods describing the generation of STING knockout and WT cell line clones in CT26.WT and BF1610 cells via the CRISPR method; 5) details of immune cell activation assays in human dendritic cells, mouse dendritic cells, NK cells and T cells analyzed by flow cytometry; 6) details regarding inoculation of BALB/C mice with A20 cells (for generation of A20 syngeneic tumors) for pharmacokinetic analysis of TAK-676 administered intravenously; 7) details regarding inoculation of BALB/C (and Goldenticket mice with BF1610 tumor cells only) mice with CT26.WT, A20, and B16F10 tumor cells for in vivo antitumor activity analysis; 8) methods describing the in vivo pharmacodynamic effects of TAK-676 by cytokine induction and immune cell activation and proliferation. The supplementary results show that TAK-676 is tolerated in syngeneic mouse models supported by Supplementary Figure 3 (percentage body weight change in BALB/C mice [CT26.WT and A20 syngeneic tumors]) and Supplementary Figure 4 (percentage body weight change in STING-deficient Goldenticket mice [STING WT and B16F10 syngeneic tumors]). [file crc-21-0161-s01.docx]

**TITLE:** TAK-676: A Novel Stimulator of Interferon Genes (STING) Agonist Promoting Durable Interferon-Dependent Anti-Tumor Immunity in Preclinical Studies

**Authors:**

Elizabeth Carideo Cunniff^1^*, Yosuke Sato^1^*, Doanh Mai^1^*, Vicky A. Appleman^1^, Shinji Iwasaki^2^, Vihren Kolev^1^, Atsushi Matsuda^2^, Judy Shi^1^, Michiyo Mochizuki^2^, Masato Yoshikawa^2^, Jian Huang^1^, Luhua Shen^1^, Satyajeet Haridas^1^, Vaishali Shinde^1^, Chris Gemski^1^, Emily R. Roberts^1^, Omid Ghasemi^1†^, Hojjat Bazzazi^1^^‡^, Saurabh Menon^1^, Tary Traore^1^^§^, Pu Shi^1^^¶^, Tennille D. Thelen^1^**, Joseph Conlon^1^^††^, Adnan O. Abu-Yousif^1^, Christopher Arendt^1^, Michael H. Shaw^1^, and Masanori Okaniwa^1^

*Authors contributed equally to this manuscript

**Affiliations:**

^1^Takeda Development Center Americas, Inc. (TDCA), Lexington, MA, USA

^2^Takeda Pharmaceutical Company, Ltd., Fujisawa, Kanagawa, Japan

^†^Current affiliation: Invicro, LLC, Needham, MA, USA

^‡^Current affiliation: Cytomx Therapeutics, South San Francisco, CA, USA

^§^Current affiliation: TScan Therapeutics, Waltham, MA, USA

^¶^Current affiliation: BeiGene, Cambridge, MA, USA

**Current affiliation: Atara Biotherapeutics, Inc., South San Francisco, CA, USA

^††^Current affiliation: Pfizer, Inflammation and Immunology, Cambridge, MA, USA

**Corresponding authors:**

Michael H. Shaw

Takeda Development Center Americas, Inc. (TDCA), 95 Hayden Avenue, Lexington, MA 02421, USA

Email: [michael.shaw2@takeda.com](mailto:michael.shaw2@takeda.com)

Phone: +1 (617) 761-6834

Masanori Okaniwa

Takeda Development Center Americas, Inc. (TDCA), 95 Hayden Avenue, Lexington, MA 02421, USA

Email: [Masanori.Okaniwa2@takeda.com](mailto:Masanori.Okaniwa2@takeda.com)

Phone: +1 (617) 444-1596

**Authors’ Disclosures**

YS, ECC, DM, VAA, SI, VK, AM, JS, MM, MY, JH, LS, SH, VS, CG, ERR, HB, SM, TT, PS, JC, AOA-Y, CWA, MHS, MO disclose employment with Takeda. OG discloses previous employment with Takeda and current employment with Invicro, LLC. TDT discloses previous employment with Takeda and current employment with Atara Biotherapeutics, Inc.

**SUPPLEMENTARY APPENDIX**

**METHODS OF** **CHEMICAL SYNTHESIS AND CHARACTERIZATION OF TAK-676**

**Preparation of TAK-676**

All commercially available reagents and solvents were used without further purification. Yields were not optimized. All reactions were monitored by thin layer chromatography (TLC) analysis on Merck Kieselgel 60 F254 plates or Fuji Silysia NH plates, or liquid chromatography with mass spectrometry (LC–MS) analysis. Nuclear magnetic resonance spectra were recorded on Bruker DPX300, Bruker AVANCE III, or Bruker Advance III plus spectrometer. Chemical shifts are given in parts per million (ppm) downfield from tetramethylsilane (δ) as the internal standard in deuterated solvent, and coupling constants (*J*) are in Hertz (Hz). Data are reported as follows: chemical shift, integration, multiplicity (s = singlet, d = doublet, t = triplet, q = quartet, quint = quintet, m = multiplet, dd = doublet of doublets, td = triplet of doublets, and brs = broad signal), and coupling constants. Analytical TLC was performed on silica gel 60 F_254_ plates (Merck) or NH TLC plates (Fuji Silysia Chemical Ltd.).

Chromatographic purification was performed on silica gel columns (Merck Kieselgel 60, 70−230 mesh size or 230−400 mesh size or Chromatorex NH-DM 1020, 100−200 mesh size) or on Purif-Pack (SI or NH, SHOKO SCIENTIFIC). LC−MS analysis was performed on Shimadzu UFLC/MS (Prominence UFLC high pressure gradient system/LCMS-2020) or Agilent LC/MS system (Agilent 1200SL/Agilent 6130MS), operating in ESI (+ or −) or APCI (+ or −) ionization mode. Analytes were eluted using a linear gradient of 0.05% trifluoroacetic acid (TFA) containing water/acetonitrile (MeCN) or 5 mM ammonium acetate containing water/MeCN mobile phase and detected at 220 nm. Preparative high-performance liquid chromatography (HPLC) was performed using L-column 2 ODS column (50 mm × 150 mm I.D) with room temperature and a flow rate of 60 mL/min. Mobile phase A and B were 5 mM ammonium acetate containing water and MeCN (9:1, volume/volume [v/v]) and 5 mM ammonium acetate containing water and MeCN (1:9, v/v), respectively. The ratio of mobile phase B was increased linearly from 42.9% to 52.9% over 9 minutes. Elemental analyses were carried out by Sumica Chemical Analysis service, Ltd., and the results were within ±0.4% of theoretical values. The purities were confirmed to be more than 95% pure as determined by analytical HPLC. The column was ZIC-HILIC (4.6 mm × 150 mm I.D.) with 40 °C and a flow rate of 1.0 mL/min. Mobile phase A and B were 5 mM ammonium acetate containing water and MeCN (9:1, v/v) and 5 mM ammonium acetate containing water and MeCN (1:9, v/v). The ratio of mobile phase B was 95% for 10 minutes, decreased linearly from 95% to 60% over 8 minutes, and 60% over the next 7 minutes, or decreased linearly from 95% to 90% over 10 minutes and 90% to 60% over the next 8 minutes, and then 60% over the 7 minutes. The analytes were detected at 254 nm. High-resolution mass spectrometry (HRMS) analysis was performed and operated in ESI (+ or −) ionization mode by Sumica Chemical Analysis service, Ltd.

**Scheme 1. Synthetic Route for Intermediate 4^a^**

*^a^*Reagents and conditions: (a) (i) pyridinium 2,2,2-trifluoroacetate, MeCN, H_2_O, rt; (ii) *t*-BuNH_2_, rt; (iii) diphenyl phosphite, pyridine, rt; (b) 1,1,1,3,3,3-hexafluoro-2-propanol, 50 °C.

**Scheme 2. Synthetic route for TAK-676^a^**

*^a^*Reagents and conditions: (a) 1,3-dichloro-1,1,3,3-tetraisopropyldisiloxane, pyridine, rt; (b) 1*H*-tetrazole, 1-methylimidazole, 2-cyanoethyl *N*,*N*,*N'*,*N'*-tetraisopropylphosphordiamidite, pyridine, MeCN, rt; (c) (i) compound **4**, 5-(ethylthio)-1*H*-tetrazole, MeCN, rt; (ii) (*E*)-*N*,*N*-dimethyl-*N'*-(3-thioxo-3*H*-1,2,4-dithiazol-5-yl)formimidamide, rt; (d) TFA, tetrahydrofuran (THF), H_2_O, 0 °C; (e) (i) 2-chloro-5,5-dimethyl-1,3,2-dioxaphosphorinane 2-oxide, pyridine, rt; (ii) 3*H*-benzo[*c*][1,2]dithiol-3-one, rt; (f) (i) MeNH_2_, methanol (MeOH), rt; (ii) HPLC separation of stereoisomers; (g) triethylamine trihydrofluoride, MeOH, triethylamine, 50°C; (h) ion exchange with BT AG 50W-X8 Resin conditioned with 1 M aqueous NaOH solution.

*(2R,3R,4R,5R)-5-(6-Benzamido-9H-purin-9-yl)-2-((bis(4-methoxyphenyl)(phenyl)methoxy)methyl)-4-fluorotetrahydrofuran-3-yl hydrogen phosphonate (****3****).* To a solution of compound **2** (10.0 g, 11.42 mmol) in MeCN (50 mL)/water (5 mL) was added pyridine 2,2,2-trifluoroacetate (2.65 g, 13.70 mmol). After being stirred at room temperature for 5 minutes, *t*-BuNH_2_ (24.10 mL, 228.33 mmol) was added to the reaction mixture. After being stirred at room temperature for 30 minutes, the solution was concentrated *in vacuo* and partitioned between water and ethyl acetate (EtOAc). The organic layer was washed with brine, dried over Na_2_SO_4_, filtered and concentrated *in vacuo*. To a solution of the residue in pyridine (100 mL) was added diphenyl phosphonate (3.29 mL, 17.13 mmol) at room temperature. The mixture was stirred at room temperature under N_2_ for 1 hour. Then water (50 mL) was added to the mixture, and it was stirred for 4 hours. The mixture was poured into water at room temperature and extracted with EtOAc. The organic layer was separated, washed with water and brine, dried over MgSO_4_ and concentrated *in vacuo.* The residue was purified by column chromatography (silica gel, eluted with 0–50% MeOH in EtOAc) to give compound **3** (7.00 g, 9.46 mmol, 83 %) as a white amorphous solid. LC–MS (ESI) m/z = 740.1 [M+H]^+^. ^1^H NMR (300 MHz, dimethyl sulfoxide-*d_6_* [DMSO-*d_6_*]) δ 3.27-3.33 (2H, m), 3.71 (6H, s), 4.19-4.32 (1H, m), 4.89-5.15 (1H, m), 5.57-5.87 (2H, m), 6.44 (1H, dd, *J* = 17.9, 1.7 Hz), 6.71-6.89 (4H, m), 7.16-7.28 (7H, m), 7.30-7.39 (2H, m), 7.48-7.70 (4H, m), 8.05 (2H, d, *J* = 7.2 Hz), 8.56 (1H, s), 8.69 (1H, s), 11.27 (1H, s). ^31^P NMR (121 MHz, DMSO-*d_6_*) δ -0.25.

*(2R,3R,4R,5R)-5-(6-Benzamido-9H-purin-9-yl)-4-fluoro-2-(hydroxymethyl)tetrahydrofuran-3-yl hydrogen phosphonate (****4****).* A mixture of compound **3** (9.9 g, 13.38 mmol) and 1,1,1,3,3,3-hexafluoro-2-propanol (25 mL, 237.45 mmol) was stirred at 50 °C under Ar for 3 hours. To the mixture was added triethylsilane (10 mL, 62.78 mmol) at room temperature. The mixture was stirred at room temperature under air for 30 minutes. The mixture was concentrated *in vacuo*. The residue was triturated in MeOH and the undissolved solid was filtered off. The filtrate was concentrated *in vacuo* and the residue was purified by column chromatography (silica gel, eluted with 20% - 50% MeOH in EtOAc in hexane) to give compound **4** (4.90 g, 11.20 mmol, 84 %) as a white solid. LC-MS (ESI) m/z 436.0 [M‒H]^‒^. ^1^H NMR (300 MHz, DMSO-*d_6_*) δ 3.62-3.84 (2H, m), 4.05-4.13 (1H, m), 4.84-5.03 (1H, m), 5.42-5.68 (1H, m), 5.80-5.89 (1H, m), 6.31-6.44 (1H, m), 6.74 (1H, *J* = 297 Hz, PH), 7.51-7.60 (2H, m), 7.61-7.70 (1H, m), 8.01-8.09 (2H, m), 8.69 (1H, s), 8.77 (1H, s), 11.26 (1H, brs). ^19^F NMR (282 MHz, DMSO-*d_6_*) δ 200.63. ^31^P NMR (122 MHz, DMSO-*d_6_*) δ 0.82.

*5-Fluoro-7-((6aR,8R,9R,9aS)-9-hydroxy-2,2,4,4-tetraisopropyltetrahydro-6H-furo[3,2-f][1,3,5,2,4]trioxadisilocin-8-yl)-3H-pyrrolo[2,3-d]pyrimidin-4(7H)-one (****6****).* Compound **5** (37.9 g, 132.88 mmol) in pyridine (760 mL) at room temperature. The mixture was stirred at room temperature under Ar for 3 hours. The mixture was concentrated *in vacuo* and the residue was partitioned between EtOAc and water. The organic layer was separated, washed with water and brine, dried over Na_2_SO_4_ and concentrated *in vacuo*. The residue was diluted with diisopropyl ether (IPE) and stirred for overnight. The precipitate was collected by filtration, washed with IPE and dried *in vacuo* to give compound **6** (32.27g, 61.1mmol, 46.0%) as a white powder. LC–MS (ESI) m/z 526.1 [M‒H]^‒^.

*2-Cyanoethyl ((6aR,8R,9R,9aR)-8-(5-fluoro-4-oxo-3H-pyrrolo[2,3-d]pyrimidin-7(4H)-yl)-2,2,4,4-tetraisopropyltetrahydro-6H-furo[3,2-f][1,3,5,2,4]trioxadisilocin-9-yl) diisopropylphosphoramidite (****7****).* Compound **6** (25.6 g, 48.51 mmol) was co-evaporated 3 times with MeCN (dry), then dissolved in N,N-dimethylformamide (dry) (110 mL). To the suspension was added 1*H*-tetrazole (3.57 g, 50.93 mmol), 1-methyl-1*H*-imidazole (2.111 ml, 26.68 mmol) and 2-cyanoethyl *N*,*N*,*N'*,*N'*-tetraisopropylphosphordiamidite (30.8 mL, 97.02 mmol) at room temperature. The mixture was stirred at room temperature under Ar overnight. The mixture was poured into sat. NaHCO_3_ aq. at room temperature and extracted with EtOAc. The organic layer was separated, washed with sat. NaHCO_3_ aq. and brine, dried over Na_2_SO_4_ and concentrated *in vacuo*. The residue was purified by column chromatography (silica gel, eluted with 30% - 60% EtOAc in hexane containing 0.5% triethylamine) to give compound **7** (26.1 g, 35.9 mmol, 74 %) as a white amorphous solid. ^1^H NMR (300 MHz, DMSO-*d_6_*) δ 0.91-1.20 (40H, m), 2.69-2.82 (2H, m), 3.51-3.72 (2H, m), 3.77-4.12 (5H, m), 4.47-4.62 (2H, m), 6.02-6.12 (1H, m), 7.14-7.22 (1H, m), 7.83-7.91 (1H, m), 12.17 (1H, brs).

*(2R,3R,4R,5R)-5-(6-Benzamido-9H-purin-9-yl)-2-((((2-cyanoethoxy)(((6aR,8R,9R,9aR)-8-(5-fluoro-4-oxo-3H-pyrrolo[2,3-d]pyrimidin-7(4H)-yl)-2,2,4,4-tetraisopropyltetrahydro-6H-furo[3,2-f][1,3,5,2,4]trioxadisilocin-9-yl)oxy)phosphorothioyl)oxy)methyl)-4-fluorotetrahydrofuran-3-yl hydrogen phosphonate (****8****).* A mixture of compound **4** (4.2 g, 9.60 mmol) and **7** (9.44 g, 12.97 mmol) was co-evaporated 3 times with MeCN. The residue was suspended in MeCN (dry) (15 mL) and THF (dry) (15 mL). 5-(Ethylthio)-1*H*-tetrazole (3.75 g, 28.81 mmol), which was co-evaporated 3 times with MeCN before the experiment, in MeCN (dry) (10 mL) was added to the mixture under Ar. After stirring at room temperature for 24 hours, (*E*)-*N*,*N*-dimethyl-*N'*-(3-thioxo-3*H*-1,2,4-dithiazol-5-yl)formimidamide (3.94 g, 19.21 mmol) was added to the mixture and the mixture was stirred for 1 hour at room temperature. The mixture was concentrated *in vacuo*. The residue was absorbed in silica gel by MeOH and purified by column chromatography (silica gel, eluted with 5% - 40% EtOAc in MeOH to give compound **8** (6.80 g, 6.20 mmol, 64.6 %) as yellow amorphous powder. The product was obtained as a mixture of stereoisomers and subjected to the next reaction without further purification. LC-MS (ESI) m/z = 1096.2 [M+H]^+^.

*(2R,3R,4R,5R)-5-(6-Benzamido-9H-purin-9-yl)-2-((((2-cyanoethoxy)(((2R,3R,4R,5R)-2-(5-fluoro-4-oxo-3H-pyrrolo[2,3-d]pyrimidin-7(4H)-yl)-4-((3-hydroxy-1,1,3,3-tetraisopropyldisiloxanyl)oxy)-5-(hydroxymethyl)tetrahydrofuran-3-yl)oxy)phosphorothioyl)oxy)methyl)-4-fluorotetrahydrofuran-3-yl hydrogen phosphonate (****9****).* To a solution of compound **8** (6.5 g, 5.93 mmol) in THF (72 mL) and water (16 mL) was slowly added TFA (16 mL, 208 mmol) at 0 °C, and the mixture was stirred for 4 hours at the same temperature. A suspension of NaHCO_3_ (25 g) in water (100 mL) was added to the mixture in an ice bath and extracted with EtOAc/THF (4:1). The organic layer was washed with brine, dried over Na_2_SO_4_, filtered and concentrated *in vacuo*. The residue was purified by column chromatography (silica gel, eluted with 20% - 50% MeOH in EtOAc) to give compound **9** (5.50 g, 4.94 mmol, 83 %). The product was obtained as a mixture of stereoisomers and subjected to the next reaction without further purification. LC–MS (ESI) m/z = 1114.2 [M+H]^+^.

*N-{9-[(5R,7R,8R,12aR,14R,15R,15aR,16R)-10-(2-Cyanoethoxy)-15-fluoro-7-(5-fluoro-4-oxo-3,4-dihydro-7H-pyrrolo[2,3-d]pyrimidin-7-yl)-16-{[3-hydroxy-1,1,3,3-tetra(propan-2-yl)disiloxanyl]oxy}-2-oxido-2-sulfanyl-10-sulfidooctahydro-12H-5,8-methanofuro[3,2-l][1,3,6,9,11,2,10]pentaoxadiphosphacyclotetradecin-14-yl]-9H-purin-6-yl}benzamide (****10****).* Compound **9** (5.5 g, 4.94 mmol) was co-evaporated twice with MeCN (dry) and pyridine (dry), respectively, before starting the experiment.

To a solution of **9** (5.5 g, 4.94 mmol) in pyridine (dry) (99 mL) was added 2-chloro-5,5-dimethyl-1,3,2-dioxaphosphorinane 2-oxide (3.19 g, 17.28 mmol) at room temperature. The mixture was stirred at room temperature under Ar for 15 minutes. To the mixture was added water (3.11 mL, 172.78 mmol) and 3*H*-benzo[*c*][1,2]dithiol-3-one (0.997 g, 5.92 mmol) at room temperature. The mixture was stirred at room temperature under air for 1 hour. The mixture was concentrated *in vacuo*. and co-evaporated twice with toluene. The residue was purified by column chromatography (silica gel, eluted with 5–40% MeOH in EtOAc) to give compound **10** (4.40 g, 3.90 mmol, 79 %) as a pale yellow amorphous powder. The product was obtained as a mixture of stereoisomers and subjected to the next reaction without further purification. LC-MS (ESI) m/z = 1128.2 [M+H]^+^.

*7-[(5R,7R,8R,12aR,14R,15R,15aR,16R)-14-(6-Amino-9H-purin-9-yl)-15-fluoro-16-{[3-hydroxy-1,1,3,3-tetra(propan-2-yl)disiloxanyl]oxy}-2,10-dioxido-2,10-disulfanyloctahydro-12H-5,8-methanofuro[3,2-l][1,3,6,9,11,2,10]pentaoxadiphosphacyclotetradecin-7-yl]-5-fluoro-3,7-dihydro-4H-pyrrolo[2,3-d]pyrimidin-4-one (****11****).* A solution of compound **10** (4.4 g, 3.90 mmol) in 40% methylamine in MeOH (20 mL, 3.90 mmol) was stirred at room temperature for 1 hour under Ar atmosphere. The mixture was concentrated *in vacuo*. The residue was purified by column chromatography (silica gel, eluted with 5–40%MeOH in AcOEt) to give 2.5 g of as a pale yellow solid. The obtained solid was purified by preparative HPLC. The combined fraction was concentrated *in vacuo* and lyophilized to give compound **11** (1.320 g, 1.359 mmol, 34.9 %). LC–MS (ESI) m/z = 971.1 [M+H]^+^. ^1^H NMR (300 MHz, CD_3_OD) δ 0.97-1.14 (24H, m), 3.20-3.22 (4H, m), 3.86-3.97 (1H, m), 4.00-4.12 (1H, m), 4.21-4.27 (1H, m), 4.30-4.43 (3H, m), 4.81-4.84 (1H, m), 5.04-5.29 (2H, m), 5.40-5.61 (1H, m), 6.28 (1H, d, *J* = 15.3 Hz), 6.39-6.51 (1H, m), 7.41 (1H, s), 7.88 (1H, s), 8.13 (1H, s), 8.28 (1H, s). ^31^P NMR (122 MHz, CD_3_OD) δ 53.8, 57.2.

*(2R,5R,7R,8R,10R,12aR,14R,15R,15aR,16R)-14-(6-Amino-9H-purin-9-yl)-15-fluoro-7-(5-fluoro-4-oxo-3,4-dihydro-7H-pyrrolo[2,3-d]pyrimidin-7-yl)-16-hydroxyoctahydro-12H-5,8-methanofuro[3,2-l][1,3,6,9,11,2,10]pentaoxadiphosphacyclotetradecine-2,10-bis(thiolate) 2,10-dioxide di-triethylamine salt (****12****).* To a solution of compound **11** (1.22 g, 1.26 mmol) in MeOH (4 mL) and triethylamine (1.00 mL) was added triethylamine trihydrofluoride (6.14 mL, 37.69 mmol) at room temperature. The mixture was stirred at 50 °C under air for 3 hours. To the mixture was added trimethylethoxysilane (58.9 mL, 376.91 mmol) at room temperature. The mixture was stirred at room temperature under air for 30 minutes and concentrated *in vacuo*. The residue was purified by column chromatography (ODS, eluted with 0–12% MeCN/10 mM triethylammonium acetate in H_2_O). The combined fraction was concentrated *in vacuo* and the residue was repeatedly diluted with deionized water and lyophilized to give compound **12** (0.870 g, 0.953 mmol, 76 %) as a white amorphous powder. LC–MS (ESI) m/z = 708.9 [M‒H]^‒^. ^1^H NMR (300 MHz, D_2_O) δ 1.17 (18H, t, *J* = 7.4 Hz), 3.09 (12H, q, *J* = 7.4 Hz), 3.94-4.08 (1H, m), 4.16-4.25 (1H, m), 4.26-4.40 (3H, m), 4.45-4.53 (1H, m), 4.74-4.78 (1H, m), 4.92-5.10 (2H, m), 5.36-5.57 (1H, m), 6.27-6.39 (2H, m), 7.24 (1H, d, *J* = 1.9 Hz), 7.89 (1H, s), 8.02 (1H, s), 8.17 (1H, s). ^31^P NMR (122 MHz, D_2_O) δ 52.1, 55.3. ^19^F NMR (282 MHz, D_2_O) δ -200.8, -164.3. HPLC purity: 99.42%.

**HPLC purity of compound 12**


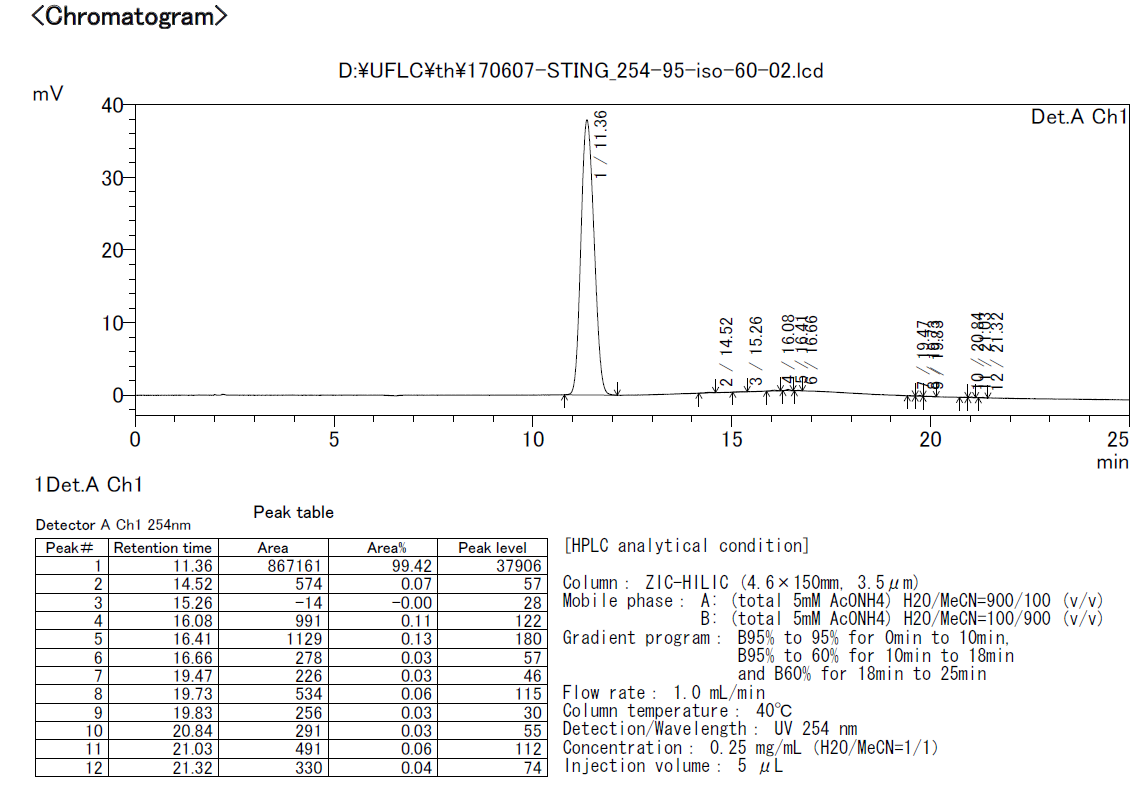


*Disodium (2R,5R,7R,8R,10R,12aR,14R,15R,15aR,16R)-14-(6-amino-9H-purin-9-yl)-15-fluoro-7-(5-fluoro-4-oxo-3,4-dihydro-7H-pyrrolo[2,3-d]pyrimidin-7-yl)-16-hydroxyoctahydro-12H-5,8-methanofuro[3,2-l][1,3,6,9,11,2,10]pentaoxadiphosphacyclotetradecine-2,10-bis(thiolate) 2,10-dioxide (****1, TAK-676****).*

Deionized water (60 mL) was passed through a column prepared by packing AG 50W-X8 cation-exchange resin (100-200 mesh, 3.9 g) in an empty column. Then, 1 M aqueous sodium hydroxide solution (36 mL) and deionized water (68 mL) were passed through the resin. Deionized water (15 mL) containing compound **12** (195 mg, 0.21 mmol) was passed through the resin after the above-mentioned pre-treatment, and deionized water (19 mL) was passed through the resin, and the obtained aqueous solution was freeze-dried to give compound **1** (165 mg, 0.21 mmol, quant.). ^1^H NMR (300 MHz, D_2_O) δ 4.02 (1H, dd, *J* = 11.7, 4.9 Hz), 4.17-4.42 (4H, m), 4.50 (1H, d, *J* = 8.3 Hz), 4.77-4.80 (1H, m), 4.90-5.11 (2H, m), 5.33-5.60 (1H, m), 6.25-6.40 (2H, m), 7.24 (1H, d, *J* = 1.9 Hz), 7.88 (1H, s), 8.00 (1H, s), 8.15 (1H, s). ^31^P NMR (122 MHz, D_2_O) δ 52.0, 55.3. ^19^F NMR ((282 MHz, D_2_O) δ -200.8, -164.3. Anal. Calculated for C_21_H_20_F_2_N_8_O_10_P_2_S_2_Na_2_・8.2 H_2_O: C, 27.96; H, 4.07; N, 12.42. Found: C, 28.08; H, 4.37; N, 12.59. HRMS *m/z* calculated for [C_21_H_23_F_2_N_8_O_10_P_2_S_2_]^+^: 711.0416. Found: 711.0390. HRMS *m/z* calculated for [C_21_H_21_F_2_N_8_O_10_P_2_S_2_Na_2_]^+^: 755.0055. Found: 755.0023. HRMS *m/z* calculated for [C_21_H_21_F_2_N_8_O_10_P_2_S_2_] ^‒^: 709.0271. Found: 709.0273. HPLC purity: 99.15%.

**^1^H NMR spectrum of TAK-676**


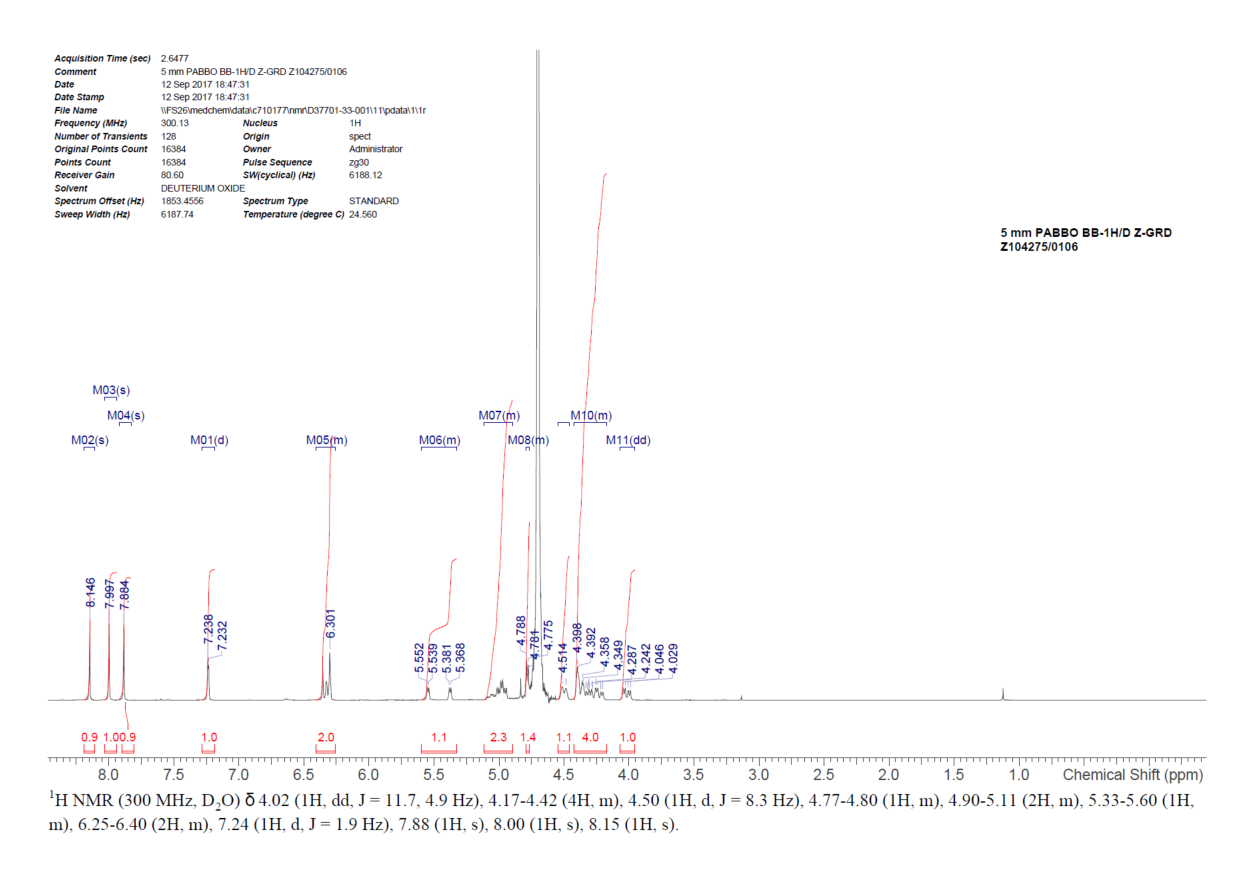


**^31^P NMR spectrum of TAK-676**


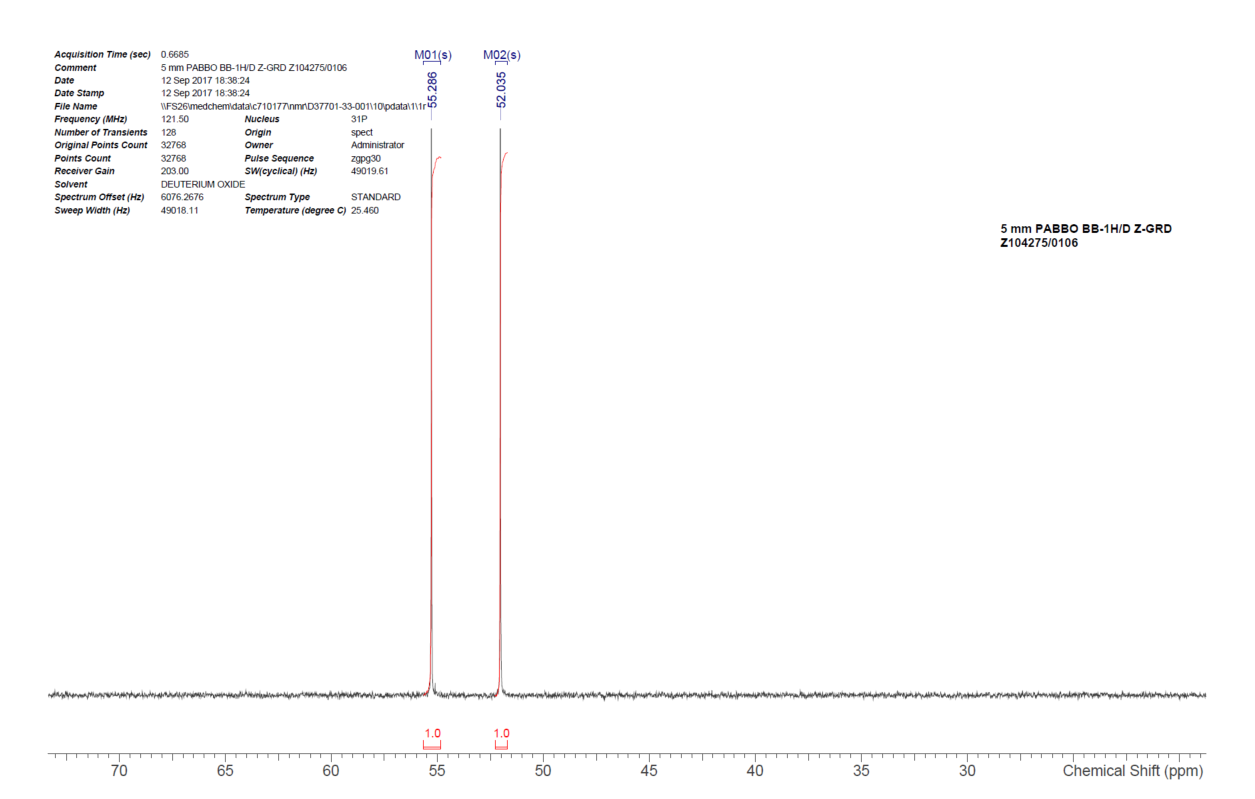


**^19^F NMR spectrum of TAK-676**


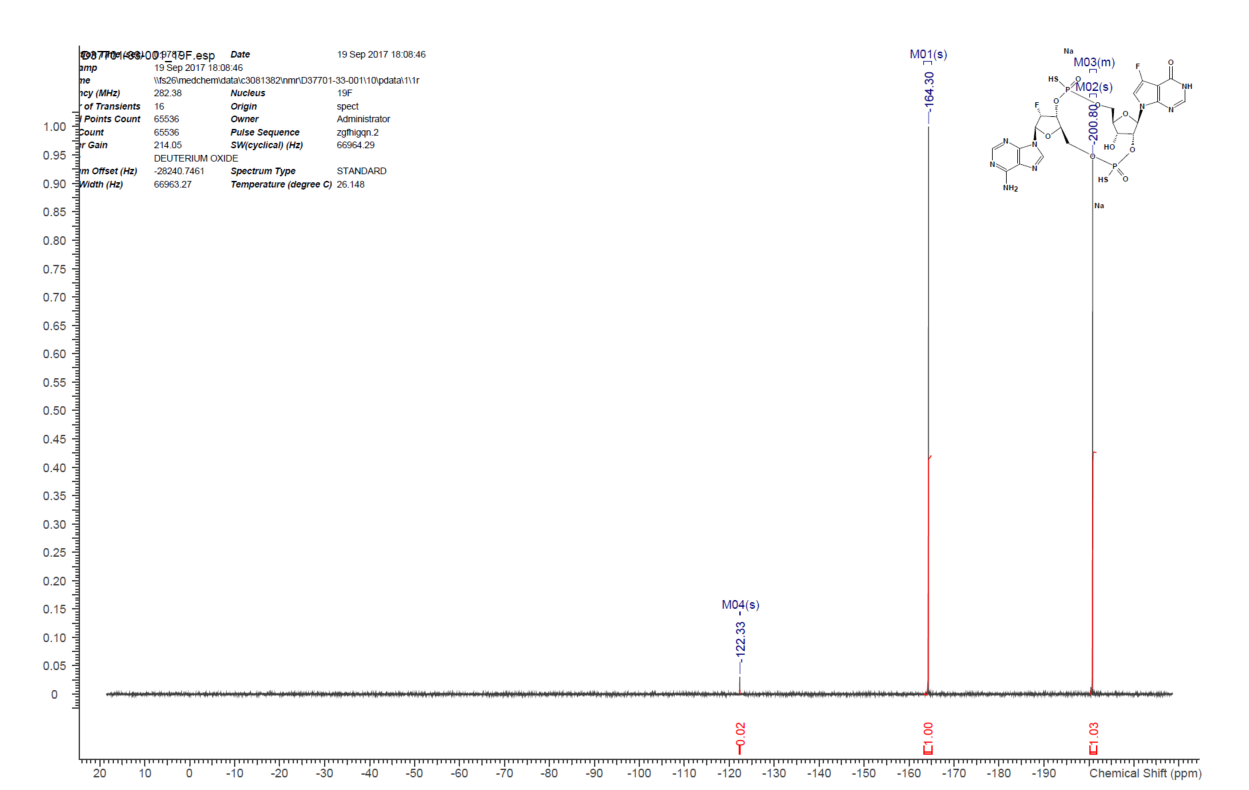


**HPLC purity of TAK-676**


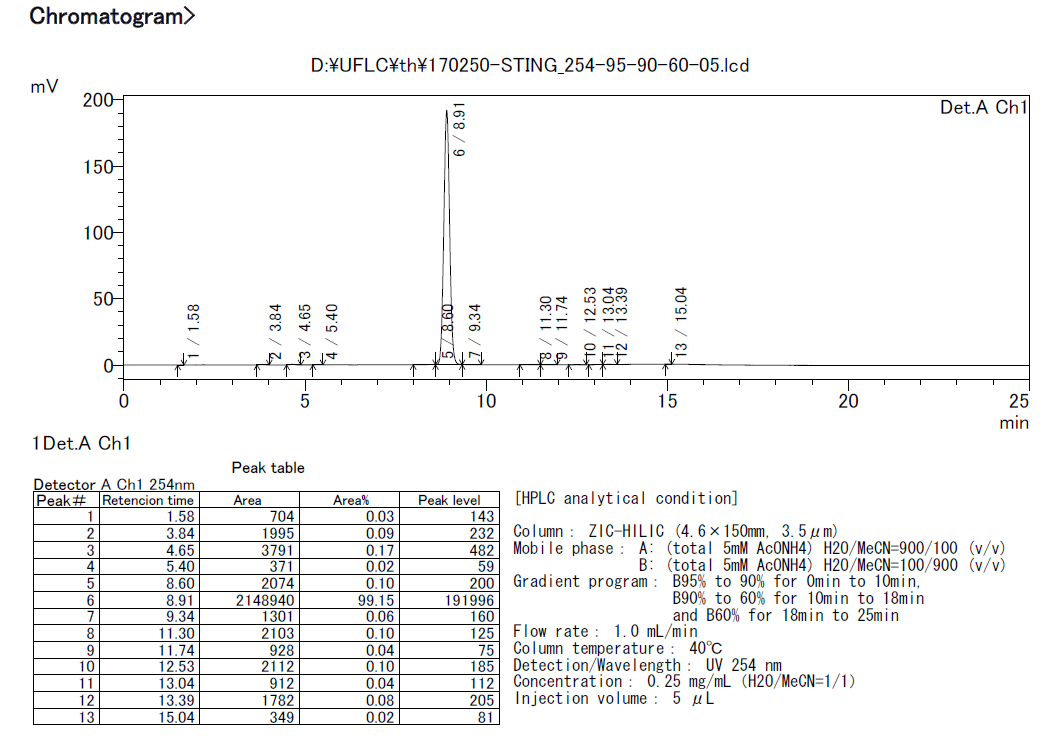


**SUPPLEMENTAL METHODS**

**Stimulator of interferon genes (STING) binding**

Briefly, mouse (mSTING) (National Center for Biotechnology Information [NCBI], National Institute of Health [Bethesda, Maryland, USA] accession number Q3TBT3) DNA encoding residues 139-378, rat STING (rSTING) (NCBI accession number F1M391) DNA encoding residues 140-379, cynomolgus monkey STING (cSTING) (NCBI accession number EHH54576.1) DNA encoding residues 140-379, and human STING (hSTING) (NCBI accession number Q86WV6) DNA encoding residues 140-379 (R232), were cloned into pET28 as N-terminal HIS and C-terminal Avi tagged fusion proteins, and a recombinant tobacco etch virus (rTEV) cleavage site between HIS tag and STING. The sequence was verified before recombinant protein expression using an *E. coli* expression system. For expression, the STING expression vector was transformed into BL21(DE3) cells (Novagen [Madison, WI, USA]). Expression was induced by adding IPTG (isopropyl β-D-1-thiogalactopyranoside) and expressed at approximately 16°C for 20 hours. STING proteins were subsequently purified by nickel affinity resin (Thermo Scientific [Sigma‑Aldrich Co.] [St Louis, Missouri, USA]), followed by TEV enzyme cleavage and Superdex 200 column (GE Healthcare [Marlborough, MA, USA]) purification. For site-directed *in vitro* biotinylation, purified Avi-tagged STING protein was incubated with Biotin (SUPELCO [Sigma‑Aldrich Co.]), ATP (Sigma‑Aldrich Co.) and BirA enzyme, followed by further purification through a Superdex 200 column (GE Healthcare). Biotinylation was confirmed by mass spectrometry analysis.

The *in vitro* assays for STING utilized the time-resolved fluorescence resonance energy transfer (TR-FRET) assay format. The detection antibody, fluorescein-cGAMP (cyclic guanine monophosphate – adenosine monophosphate) and other reagents were purchased, and the assays were performed as follows: 40 nL of compounds in dimethyl sulfoxide (DMSO) were added to wells of white, 384-well microtiter plates (Greiner 784075). 2 μL of STING assay buffer (phosphate buffered saline [PBS] and 0.01% bovine serum albumin [BSA]) containing fluorescein-labeled ligand (c[G(2',5')p-2'-Fluo-AHC-A(3',5')p]- Biolog C 195, 100 nM final) and terbium (Tb)-labeled Streptavidin (Streptavidin-Tb cryptate-CisBio 610SATLB) were added. Then, 2 μL of STING assay buffer containing STING protein (100 nM final) was added and the mixture was incubated at room temperature for 60 minutes.

The plates were then read on a BMG PheraStar Plus reader (LanthaScreen module, BMG Labtech [Ortenberg, Germany]). This assay format is based on the displacement of the fluorescein-labeled ligand by compound. The extent of displacement of the fluorescein ligand results in a decrease of the emission ratio. The half maximal inhibitory concentration (IC_50_) titration for TAK‑676 against the various STING orthologs were run below the equilibrium dissociation constant (K_d_) of the tracer to ensure maximum sensitivity.

For the assay method described above, the test compound decrease of the emission ratio, at various concentrations, was calculated relative to untreated and control-treated samples. Compound concentration versus decrease of the emission ratio curves were fitted to generate IC_50_ values.

**Pathway activation assays**

*THP1-Dual™ and CT26.wild type (WT) cell lines*

THP1-Dual™ human tumor cells were cultured in Roswell Park Memorial Institute (RPMI) 1640 media supplemented with 10% heat-inactivated fetal bovine serum (FBS), 10 µg/mL blasticidin, 100 µg/mL zeocin, 100 µg/mL normocin, and 100 µg/mL Penicillin-Streptomycin (Pen-Strep). CT26.WT and STING deficient CT26.KO tumor cells were cultured in RPMI 1640 media supplemented with 10% FBS. All cells were maintained at approximately 37°C in a humidified cell culture chamber containing 5% CO_2_.

To determine the activity of TAK-676 on the STING pathway, cells were plated into 6-well tissue culture plates at a density of 1.8 × 10^6^ cells per well for THP1-Dual™ and 0.8 × 10^6^ cells per well for CT26.WT or CT26.KO. Following overnight incubation in a humidified 37°C /5% CO_2_ incubator, cells were treated with TAK-676 at multiple concentrations or DMSO as a control and placed in a humidified 37°C /5% CO_2_ incubator for 3 hours.

For the human THP1-Dual™ cell line, 1.8 × 10^6^ cells were treated with DMSO or TAK-676 (10, 3.3, and 1.1 µM) for 3 hours. For the mouse cell line (CT26.WT), 0.8 × 10^6^ cells were treated with DMSO or TAK-676 (0.3, 1.1, 3.3, and 10 µM) for 3 hours. For experiments in STING deficient CT26.KO cell lines, DMXAA (50 µg/mL) was used as a control for a treatment time of 1 hour prior to cell lysis and blotting.

After stimulation of THP1-Dual™ cells with TAK-676, medium and cells were collected by pipetting into 15 mL tubes and centrifuged at 800 relative centrifugal force (RCF) for 5 minutes in a Beckman Coulter Allegra™ 6R centrifuge and washed once in ice-cold PBS. Cells were spun again for 3 minutes, PBS was removed by vacuum suction, and pellets were frozen at approximately −80°C. Cell pellets were lysed in 1% Triton X-100 lysis buffer (10% glycerol, 20 mM Tris-HCl pH 7.5, 150 mM NaCl, 1 µM EDTA [ethylenediaminetetraacetic acid], 1 µM DTT [dithiothreitol], 0.4 µL/mL [100 U/mL lysis buffer] Benzonase^®^ Nuclease) containing protease and phosphatase inhibitor cocktails.

After stimulation of CT26.WT or CT26.KO cells with TAK-676, the medium was removed and transferred to a 15 mL conical tube and stored on ice. 1 mL of ice-cold PBS was then added to the cells and swirled to ensure full coverage of the adherent cells on the well bottom. Using a cell scraper, the cells were scraped to remove them from the plate. Scraped cells and PBS were collected into the corresponding medium-containing 15 mL conical tubes and kept briefly on the ice. Tubes were spun in a centrifuge (Sorvall™ Legend™ XTR) to pellet cells at 83 RCF for 5 minutes. PBS was removed from the cell pellet by aspiration. 1 mL of fresh, cold PBS was added to the pellet. Cells were then re-suspended in the PBS and transferred to microcentrifuge tubes. The cells were spun in a refrigerated microcentrifuge (Eppendorf 5417R) at 106 RCF set at 4°C, for 5 minutes, PBS was removed by aspiration, and cell pellets were immediately frozen at approximately −80°C. Cell pellets were lysed in radioimmunoprecipitation assay buffer (RIPA) which comprised 50 mM Tris-HCl pH 7.5, 150 mM NaCl, 1% nonyl phenoxypolyethoxylethanol (NP40) detergent, 1% sodium deoxycholic acid (DOC), and 1 mM EDTA. Immediately before use, the RIPA buffer was supplemented with 1 mM phenylmethylsulfonyl fluoride (PMSF), 50 mM sodium fluoride (NaF), 50 mM sodium orthovanadate, 25 mM beta-glycerophosphate, complete EDTA-free protease inhibitor tablet, and 2 µL/mL Benzonase^®^ Nuclease (500 U/mL lysis buffer).

Cell lysates from both the THP1-Dual™, CT26.WT, and CT26.KO cells were cleared of insoluble debris by centrifuging at 16,000 RCF in a microcentrifuge for 10 minutes in a 4°C cold room. The protein concentrations of the lysates from the cells were determined by Bradford and bicinchoninic acid assays, respectively, using BSA standards. Lysates were denatured in NuPAGE™ lithium dodecyl sulfate (LDS) sample buffer containing DTT (50 mM final concentration) as a reducing agent at 95°C for 5 minutes and approximately 70°C for 10 minutes for THP1-Dual™ and CT26.WT or CT.26 KO cells, respectively. Denatured lysates were resolved on NuPAGE™ 4-12% Bis-Tris gels in MES/SDS (2-(*N*-morpholino) ethanesulfonic acid/SDS) running buffer and transferred to PVDF (polyvinylidene difluoride) membranes using a TE70XP semi-dry transfer apparatus (Hoefer, Inc. [Holliston, MA, USA]) according to the manufacturer’s instructions.

For the THP1-Dual™ cell line, membranes were blocked for 1 hour in tris-buffered saline solution containing 1% tween detergent (TBST) containing non-fat dry milk (5%) with gentle agitation. For the CT26.WT and CT26.KO cell line, membranes were rinsed once with distilled water and transferred to dishes containing Odyssey^®^ Blocking Buffer and then agitated at room temperature for 1 hour to block the membranes.

Following blocking, membranes were probed overnight with primary antibodies at 4°C with constant agitation. The following primary antibodies were used for the THP1-Dual™ human cell line at a 1:1000 dilution in TBST containing BSA (5%) at 4°C with constant agitation unless otherwise indicated: phospho-TBK1 serine (S) 172 (S172, 1:2000 dilution), total TBK1, phospho-STING S366, total STING, phospho-IRF3 S396, total IRF3, and glyceraldehyde 3-phosphate dehydrogenase (GAPDH) (1:3000 dilution). The following primary antibodies were used for CT26.WT and CT26.KO murine cell lines at a 1:1000 dilution in Odyssey^®^ Blocking Buffer unless otherwise indicated: phospho-TBK1 S172 (1:2000 dilution), TBK1 (1:2000 dilution), phospho-STING S366 STING (1:2000 dilution), phospho-IRF3 S396 IRF3, and alpha Tubulin (1:10,000 dilution).

After the overnight primary antibody probe, membranes were washed 3 times in TBST and probed with secondary antibody at room temperature with constant agitation in Odyssey^®^ Blocking Buffer. The secondary antibody probe used for the THP1-Dual™ cell line was Alexa Fluor™ 680 goat anti-rabbit immunoglobulin (Ig)G or Alexa Fluor™ 680 goat anti-mouse IgG at a 1:6000 dilution for 90 minutes. For the CT26.WT and CT26.KO cell lines, it was IRDye 680RD goat anti-rabbit IgG (H+L) (1:4000 dilution) and IRDye 800CW goat anti-mouse IgG (H+L) (1:4000 dilution) for 1 hour. After extensive washing in both cell lines, membranes were scanned using an ODYSSEY^®^ CLx infrared imaging system.

After the overnight probe in the human THP1-Dual™ cell line, the phospho-STING S366 membrane was washed in TBST and probed with HRP-conjugated secondary antibody for 90 minutes in TBST with 5% non-fat dry milk with constant agitation. This membrane was washed and developed using ECL reagent and X-ray film on a Kodak M35A X-OMAT Processor.

*HEK293T cell line*

As HEK293T parental cells lack endogenous STING expression, they are well suited for transient transfection leading to overexpression of WT and STING isoforms, and also as a negative control for the assay. A stable HEK293T cell line expressing pNL[NLucP/ISRE/Hygro]vector (Promega [Fitchburg, WI, US]) was established as per the manufacturers protocol at Shonan Research Center (SRC) (Takeda Pharmaceutical Company Limited [Fujisawa, Kanagawa, Japan]) and transferred to Millennium. The cells were cultured in growth media (DMEM [Dulbecco’s Modified Eagle Medium]) with 10% FBS and transiently transfected with STING DNA from human WT (R232), or other variants [R232H, R293Q, G230A-R293Q (AQ), and R71H-G230A-R293Q (HAQ)] that exist in human populations (14) the day before the assay using forward transfection assisted with Fugene^®^HD (Promega, Catalog No. E2312). The cells were plated into a white, Corning 384 well plate at 5000 cells/25 µL per well density in growth media. For each compound plate, 2 plates of cells transiently expressing the human STING were plated. On the day of the experiment, the media for 1 plate of cells was replaced by 25 µL/well digitonin buffer (50 mM HEPES pH 7.0, 100 mM KCl, 3 mM MgCl_2_, 0.1 mM DTT, 85 mM sucrose, 0.2% BSA, 0.1 mM adenosine triphosphate [ATP], 0.1 mM guanosine triphosphate [GTP], and 10 µg/mL digitonin), followed by dosing with 62.5 nL of test compound. The cells were incubated for 30 minutes at 37°C and 5% CO_2_ to permeabilize the cell membranes. The digitonin buffer was then replaced by 25 µL cell growth media, and the cells were incubated at 37°C and 5% CO_2_ for 4 hours. At the end of the incubation, 15 µL/well of the Nano-Glo^®^ Detection System N1150 (Promega) was added, and luminescence was measured immediately using the LEADseeker™ Imaging System (General Electric Healthcare). For the other cell plate that had not been permeabilized with digitonin, 62.5 nL of test compound in DMSO was added to each well using the Labcyte ECHO 555. These plates were incubated at 37°C 5% CO_2_ for 20 hours before the Nano-Glo^®^ Detection System was added and the luminescence detection was measured immediately as described above.

*THP1-Dual™ cells*

THP1-Dual™ cells (InvivoGen, ID thpd-nfis) were obtained from InvivoGen (San Diego, CA, US). These cell lines were derived at InvivoGen from the human THP-1 acute myelocytic leukemia (AML) cell line by stable integration of the Lucia luciferase gene, a secreted luciferase reporter gene, under the control of an ISG54 minimal promoter in conjunction with 5 interferon (IFN)-stimulated response elements. On the day of the experiment, the cells were plated into black, 384-well plates at 7500 cells/25 µL per well density in growth media (RPMI 1640, 2 mM L-glutamine, 25 mM HEPES (4-(2-Hydroxyethyl) piperazine-1-ethanesulfonic acid, N-(2-Hydroxyethyl)piperazine-N′-(2-ethanesulfonic acid), 10% heat-inactivated FBS, 100 μg/mL Normocin™, 100 units (U)/mL-100 μg/mL Pen-Strep, 10 μg/mL of blasticidin, and 100 μg/mL of Zeocin). The cell plates were dosed with 62.5 nL of the testing compounds using the Labcyte ECHO 555, and then incubated at 37°C, 5% CO_2_ for 20 hours. At the end of the incubation, 15 µL/well of the QUANTI-Luc™ (InvivoGen, ID rep-qlc1) were added, and luminescence was measured immediately using the LEADSeeker™ Imaging System.

*RAW-Lucia™ ISG cells*

RAW-Lucia™ interferon-stimulated genes (ISG) cells (InvivoGen, ID rawl-isg) were derived by InvivoGen from the mouse RAW 264.7 macrophage cell line by stable integration of the Lucia luciferase gene, a secreted luciferase reporter gene, under the control of an ISG54 minimal promoter in conjunction with 5 IFN-stimulated response elements. One day before the experiment, the cells were plated into a white, 384-well plate (Corning, Catalog No.356661) at 12500 cells/25 µL per well density in growth media (DMEM, 2 mM L-glutamine, 4.5 g/L glucose, 10% FBS, 100 μg/ml Normocin™, 50 U/mL-50 μg/mL Pen-Strep, and 200 μg/mL of Zeocin). For each compound plate, 2 plates of cells were plated. The cells were incubated at 37°C overnight. On the day of the experiment, cell growth media from 1 of the 2 cell plates were replaced with 25 µL/well digitonin buffer (50 mM HEPES pH 7.0, 100 mM KCl, 3 mM MgCl_2_, 0.1 mM DTT, 85 mM Sucrose, 0.2% BSA, 0.1 mM ATP, 0.1 mM GTP, and 10 µg/mL digitonin), followed by dosing with 62.5 nL of test compound. The cells were incubated for 30 minutes at 37°C to permeabilize the cell membranes. The digitonin buffer was then replaced by 25 µL cell growth media, and the cells were incubated at 37°C 5% CO_2_ for 6 hours. At the end of the incubation, 10 µL/well of the QUANTI-Luc™ (InvivoGen, ID rep-qlc1) were added, and luminescence was measured immediately using the LEADseeker. For the cell plate that was not treated with digitonin, 62.5 nL of the test compound in DMSO was added to the cells, and incubated at 37°C, 5% CO_2_ for 20 hours before 10 µL/well of the QUANTI-Luc was added and the luminescence detection was measured immediately as described above.

**Generation of STING knockout and WT cell line clones in CT26.WT and B16-F10 cells**

TrueGuide Synthetic guide RNAs (gRNA) were obtained from Thermo Fisher Scientific and dissolved in RNAse-Free water. CRISPR knockout efficiency of five different mSTING gRNAs was assessed and the GFP_sg_IVT_gRNA guide was used as a negative control. gRNAs were evaluated at a final concentration of 1 μg/μL. The following reagents were used for CRISPR knockout by *in vitro* transfection and were obtained from Thermo Fisher Scientific: Cas9 Nuclease V2 (100 μL, 5 μg/μL) (Cat. no. A27865V), Cas9 PLUSTM Reagent (200 μL) (Cat. no. 100035635), Lipofectamine® CRISPRMAXTM (Cat. no. 100035630), and Opti-MEM™ I Reduced Serum Medium (Cat. no. 31985).

Cells were plated into 6-well dishes and incubated overnight at 37°C with 5% CO_2_. Transfections were performed in duplicate to allow for cell harvesting and analysis at 48 and 72 hours post-transfection. Western blotting for mSTING protein and PCR analysis of mSTING genomic DNA was performed to determine the relative knockout efficiency of the guide RNAs. mSTING/TMEM173 sg-3: *GCTCTTCAGCCAGACAGCAG* and mSTING/TMEM173 sg-4: *GTACCCAATGTAGTATGACC* were selected as optimal guide sequences.

Once optimal guide sequences were determined the method described above was repeated for the two selected gRNAs. At 48 hours post-transfection, cell pools were collected and prepared for limited dilution cloning. Briefly, medium was removed from the pools, cells were washed twice with 1 mL of pre-warmed PBS, 160 μL of .25 % Trypsin-EDTA was added to each well; cells were incubated in trypsin at 37°C for 2 minutes, and cells were collected and dispersed by pipetting up and down in 2 mL of pre-warmed medium. Pools were counted using the CellometerTM Auto T4 automated cell counter and then repeatedly diluted 1:10 in media, each time recounting to ensure accuracy. Upon attaining a cell suspension of 4 cells/mL, this dilution was plated into five 96-well tissue culture plates at 200 μL/well so as to arrive at 0.8 cells/well. Plates were incubated for 96 hours at 37°C with 5% CO_2_. Over the following days and weeks, all 96-wells were scanned by eye using a light microscope. Single colonies were identified and marked. Single colonies were selected and expanded into 6-well dishes and then into 10 cm^2^ dishes. Confluent clones were harvested and evaluated by Western blotting for mSTING protein and PCR analysis of mSTING genomic DNA to reconfirm efficient knock down. Several confirmed clonal mSTING knockouts, as well as matched WT controls shown to have intact mSTING, were expanded, banked, tested for murine pathogens, and cryopreserved for use in subsequent *in vitro* and *in vivo* studies

**Immune cell activation assays**

*Human DC activation assay*

Human peripheral blood mononuclear cells (PBMCs) from 5 healthy donors were obtained from STEMCELL Technologies Inc. (Cambridge, MA, USA) and Precision Medicine Group, Inc. (Frederick, MD, USA).

CD14-positive (+) monocytes were isolated from PBMCs using human CD14 microbeads (Miltenyi Biotec) according to manufacturer’s instructions. Briefly, total live cell numbers were determined using cell counter Vi-CELL XR (Beckman Coulter. Inc.) and then cells were pelleted by centrifugation at room temperature at 350 × g for 10 minutes. Cell pellets were resuspended in 80 µL of MACS buffer (PBS with 2% FBS) per 10^7^ total cells and then 20 µL of CD14 microbeads per 10^7^ total cells were added and incubated for 15 minutes in the refrigerator after mixing well. CD14-labeled cells were then washed with 2 mL of MACS buffer per 10^7^ total cells and centrifuged at room temperature at 350 × g for 10 minutes. After centrifugation, the supernatants were aspirated off completely, then the cell pellets were resuspended up to 10^8^ total cells in 500 µL of MACS buffer. LS Columns (Miltenyi Biotec) were placed in the magnetic field of the MidiMACS™ Separator (Miltenyi Biotec) and then columns were rinsed with 3 mL of MACS buffer. After the column reservoir emptied, the labeled PBMC suspension was loaded onto the column. The columns were washed 3 times with 3 mL of MACS buffer, the column was removed from the separator, and then placed in a 15 mL conical tube. Five mL of MACS buffer was pipetted onto the column and the bound cells were immediately flushed out by firmly pushing the plunger into the column. On Day 1, after CD14+ cell numbers were determined using Vi-CELL XR, cells were plated in DC medium RPMI 1640 media supplemented with 10% heat-inactivated human AB serum (Corning Incorporated), 2 mM L-Glutamine (Thermo Fisher Scientific, Inc.), 1 × minimum essential medium (MEM) non-essential amino acid (NEAA) (Thermo Fisher Scientific, Inc.), 1 mM sodium pyruvate (Thermo Fisher Scientific, Inc.), 0.5 mmol/L monothioglycerol solution (Wako Pure Chemical Industries, Ltd), and 100 U/mL Pen-Strep (Thermo Fisher Scientific, Inc.) with 100 ng/mL recombinant human granulocyte macrophage-colony stimulating factor (GM-CSF) (PeproTech, Inc.) and 50 ng/mL recombinant human interleukin (IL)-4 (Invitrogen). Half the volume of the medium was replaced with fresh medium that contained GM-CSF and IL-4 on Day 3 and Day 5.

Floating cells, comprising human monocyte-derived DCs (MoDCs), were collected on Day 6 and cell numbers were determined using Vi-CELL XR. MoDCs were plated in 96-well U-bottom plates at a density of 1.6 × 10^5^cells in 90 µL of DC medium. Immediately after seeding, the cells were treated with 0.03 µM, 0.1 µM, 0.3 µM, 1 µM, 3 µM, 10 µM, or 30 µM of TAK-676, or DMSO as a control for 24 hours in a humidified, 5% CO_2_ incubator, set at 37°C, before performing the assay. The final volume was 100 µL for each well.

The 96-well U-bottom plates with compound treated cells were centrifuged for 10 minutes at 350 × g set at 4°C. Then the supernatant was removed, and pelleted cells were washed once with PBS. The cells were resuspended in PBS containing the LIVE/DEAD™ Fixable Near-IR Dead Cell Staining solution (Thermo Fisher Scientific, Inc.) diluted 1:1000 and incubated at room temperature for 10 minutes. After incubation, the cells were centrifuged and supernatant was discarded, and cells were washed once with stain buffer (BSA) (Becton, Dickinson, and Company [Franklin Lakes, NJ, USA]). Cells were then resuspended in stain buffer (BSA) (Becton, Dickinson, and Company) containing IgG from human serum (Sigma-Aldrich, Inc.) diluted 1:500 and incubated on ice for 20 minutes. The cells were stained on ice for 20 minutes while being protected from light. After staining, the cells were pelleted for 10 minutes at 350 × g set at 4°C. The cells were resuspended with stain buffer. Data for each sample was acquired by a LSR Fortessa™ flow cytometer (Becton, Dickinson, and Company) with FACS Diva software™, Version 8.0.1 (Becton, Dickinson, and Company).

Flow cytometric data files were analyzed using the FlowJo^®^ software, Version 10.1 (FlowJo, LLC [Ashland, OR, USA]). The threshold between positive and negative cells was determined using the unstained controls. MoDCs were defined as LIVE/DEAD™ Fixable Near-IR Dead Cell Staining Dye negative, human leukocyte antigen (HLA)-DR isotype+, CD14 negative, CD11c+, CD45+, CD209+ population. The mean fluorescence intensity (MFI) of DCs expressing maturation marker CD86 and MoDC viability, which was defined as LIVE/DEAD™ Fixable Near-IR Dead Cell Staining Dye negative population from the parent gate, was recorded and graphed using the GraphPad Prism software, Version 7.03 (GraphPad Software, Inc. [La Jolla, CA, USA]).

*Mouse DC activation assay*

Two BALB/c mice were sacrificed and dissected on Day 0 to obtain both femurs and tibiae without cutting the bone ends. The collected bones were cleaned from all the attached tissues by tissue paper and sterilized by brief immersion in 70% ethanol for 10 minutes. The bones were then transferred to a PBS-filled cell culture dish and incubated for additional 5 minutes. The ends of the bones (epiphyses) were cut, and the bones were infused with 5 mL of BMDC Medium 1 (RPMI 1640 medium with 1% Pen-Strep, serum free) using a sterile syringe with a 27G needle (Becton, Dickinson and Company) on the cell culture dish. The medium containing all cells was collected in a 50 mL conical tube (Thermo Fisher Scientific, Inc.) and then spun in a centrifuge at 1100 rpm, set at 4^o^C for 10 minutes. The cell pellet was washed once with BMDC Medium 1 and centrifuged again with the same setting. After washing, the pellet was resuspended in 2 mL of ACK Lysis Buffer (Thermo Fisher Scientific, Inc.) and incubated for 5 minutes at room temperature for red blood cell (RBC) lysis. After a 5-minute lysis, 25 mL of BMDC Medium 1 was then added to the cell suspension to neutralize the RBC lysis process. The cell suspension was then spun in a centrifuge at 1100 rpm, set at 4^o^C for 10 minutes, and washed once more with BMDC Medium 1. After the washing, the cells were resuspended in 10 mL of BMDC Medium 2 (RPMI 1640 medium with 10% heat-inactivated FBS and 1% Pen-Strep) and counted using a cellometer (Nexcelom Bioscience, LLC) for determining cell density. After counting, the cells were diluted to 2 × 10^5^ cells/mL with BMDC Medium 2 supplemented with GM-CSF (final concentration 20 ng/mL) (Sigma-Aldrich [St. Louis, MO, USA]) and cultured at 2 mL/well in a 6-well culture plate (Corning Incorporated [Corning, NY, USA]) with 5% CO_2,_ in a cell-culture incubator (Thermo Fisher Scientific, Inc. [Waltham, MA, USA]) set at 37°C. On Day 3, another 2 mL of BMDC Medium 2 supplemented with 20 ng/mL GM-CSF was added into the cell culture wells. On Days 6 and 8, half of the medium was removed from the wells and spun in a centrifuge at 1100 rpm, set at 4°C for 10 minutes. The pellet was then resuspended with the same amount of fresh BMDC Medium 2 supplemented with 10 ng/mL GM-CSF and added back to the wells. On Day 10, the differentiated floating DCs were collected by gently pipetting up and down, centrifuged, and resuspended with cold BMDC Medium 1 to minimize maturation. The yield of BMDC differentiation was determined by cell counting using a cellometer (Nexcelom Bioscience LLC. [Lawrence, MA, USA]).

The differentiated immature DCs were plated in 96-well plates (Corning Incorporated [Corning, NY, USA]) at a density of 2.5 × 10^5^ cells/well. GP-100 antigen peptides (final concentration 10 µg/mL) were added to each well. After the addition of GP-100 peptides, TAK-676 was added to each well at final concentrations of 0 (vehicle control, DMSO), 0.1, 0.3, 1, 3, and 10 µM. The plates were then placed into the cell incubator. After various incubation periods (1, 3, 6, 24, 48, or 72 hours), the floating mature bone marrow DCs (BMDCs) were collected by pipette and washed twice with cold fluorescence-activated cell sorting (FACS) stain buffer (BD Biosciences [San Jose, CA, USA]) in V-bottom 96-well plates (Corning Incorporated). After washing, the cells were stained with CD11c-BV605, CD8a-PE (DC markers) and CD86-APC (DC activation marker) mouse antibodies at 4°C for 20 minutes. After antibody staining, the cells were washed and resuspended in FACS stain buffer containing live and dead dye, 7‑aminoactinomycin D (7‑AAD) (Thermo Fisher Scientific, Inc. [Waltham, MA, USA]). After a 10-minute incubation, the stained cells were analyzed with a BD FACSCelesta flow cytometer (BD Biosciences) and the data were recorded using BD FACSDiva™ software (BD Biosciences). Each timepoint and TAK‑676 concentration was conducted in duplicate.

Flow cytometric data files were analyzed using the FlowJo^®^ software, Version 10.4 (FlowJo, LLC). Data were gated on 7-AAD negative, CD11c+ and CD8a+ populations to include only live CD11c+ CD8a+ mouse BMDCs. The cell viability and MFI of DCs expressing maturation marker CD86 were exported to Microsoft Excel 2016 (Microsoft [Redmond, WA, USA]) and graphed with GraphPad Prism 7.04 (GraphPad Software, Inc. [La Jolla, CA, USA]). The half maximal effective concentration (EC_50_) value of TAK-676 BMDC activation was fitted using CD86 MFI values at the 24-hour timepoint by GraphPad Prism 7.04 (GraphPad Software, Inc. [La Jolla, CA, USA]).

*NK and T cell activation*

For measuring TAK-676-mediated induction of CD69 on NK and T cells, 225 µL of human whole blood from 5 healthy donors was pipetted separately into each well of 96-well round bottom plates. Immediately after pipetting, blood samples were treated with 0.01, 0.03, 0.1, 0.3, or 1 µM TAK-676 or DI water as the vehicle control for 24 hours in a humidified 37°C, 5% CO_2_ incubator before performing the analysis.

100 µL of TAK‑676-treated whole blood was pipetted into a deep-well, 96-well, v-bottom plate (ThermoFisher [Waltham, MA, USA]), and 1.8 mL of 2 × RBC lysis buffer (BD Pharmlyse buffer [BD Biosciences (San Jose, CA, USA)]) was added to each well and incubated at 37°C for 15 minutes. After incubation, plates were centrifuged for 5 minutes at 500 × g, and the cells were washed once with stain buffer (BD Biosciences [San Jose, CA, USA]) and lysed once more for 10 minutes at room temperature. After incubation, the plates were centrifuged for 5 minutes at 500 × g and the cells were washed once with stain buffer. To stain cell-surface markers, the cell pellet was resuspended in 100 µL of stain buffer with staining antibody CD3-fluorescence isothiocyanate (FITC), CD4-allophycocyanin (APC), CD8-AlexaFluor 700 (T-cell markers), CD16-Brilliant Violet 650™ (BV650) and CD56- phycoerythrin (PE) (NK-cell markers), and CD69-BD Horizon™ (BV786) brilliant stain (activation marker) at 4°C for 20 minutes while being protected from light. After incubation, plates were centrifuged for 5 minutes at 500 × g and then cells were washed twice with stain buffer. The cell pellet was resuspended in 200 µL of stain buffer containing 7-AAD live and dead dye (Thermo Fisher Scientific, Inc [Waltham, MA, USA]) and incubated at 4°C for 10 minutes. The data were collected by a BD FACSCelesta™ Flow Cytometer (BD Biosciences [San Jose, CA, USA]) and analyzed with FlowJo^®^ software, Version 10.1 (FlowJo, LLC [Ashland, Oregon, USA]).

For the flow cytometry analysis, the cells were first gated on lymphocytes (side scatter area [SSC-A] versus forward scatter area [FSC-A]) and singlets (forward scatter height [FSC-H] versus FSC-A for doublet discrimination). The singlet gate was further analyzed for the uptake of 7‑AAD to determine live versus dead cells and their expression of CD3 or CD16, considering only the live T- or NK-cell population. The surface expression of CD69 on CD4+ and CD8+ T cells or CD16+ CD56+ NK cells was then determined from the gated population.

Data were graphed using GraphPad Prism software, Version 7.04 (GraphPad Software, Inc. [La Jolla, CA, USA]). The EC_50_ and Hill slope were estimated using GraphPad Prism Version 7.04, using an agonist versus response model with variable slope. The *in vitro* blood-to-plasma ratio of TAK‑676 in human blood was estimated to be 0.73, a mean of the values obtained from testing concentrations of 0.1, 1, and 10 µM (Strab R. Human, rat, mouse, dog, and monkey blood to plasma partitioning. Exton [PA, USA]: Absorption Systems LLC; 2018. Report 18MILLP1.). This ratio was then applied to estimate the plasma concentration-based EC_50_ by dividing the blood-based concentration EC_50_ by 0.73 (*in vitro* blood/plasma ratio). The CD8 T-cell data for Donor 2 was not adequately characterized by the default fitting model. Therefore, the parameters for Donor 2 were estimated by constraining the Hill slope to a value of 2.97. This value represents the mean value of the Hill slope from the other 4 donors.

**Pharmacokinetics of TAK-676 in female BALB/C mice bearing A20 syngeneic tumors**

Six- to 7-week-old female BALB/c mice were inoculated SC with 1.0 × 10^6^ A20 tumor cells in the right flank. When tumors grew to approximately 300 to 800 mm^3^, animals were assigned into groups (*n* = 3/time point). Each group of animals received a single TAK-676 administration (at 0.025, 0.125, 0.25. 0.5, and 2 mg/kg) via IV dosing. Doses were calculated based on a body weight of 23 g. Animals were sacrificed in accordance with Institutional Animal Care and Use Committee (IACUC) guidelines at defined timepoints (5, 10 and 30 minutes, and 1, 3, 24, and 72 hours post dose), and tumor and plasma samples were harvested according to approved research operating procedures (ROPs).

TAK-676 was formulated as a clear solution in PBS at multiple dose concentrations. Each animal was administered the TAK-676 formulation by IV injection into the lateral tail vein.

For plasma collection, approximately 500 µL of whole blood was obtained via cardiac puncture, placed into tubes coated with dipotassium ethylenediaminetetraacetic acid (K_2_EDTA) to prevent clotting, and centrifuged at 10,000 rpm for 5 minutes. Approximately 200 µL of plasma was then transferred into 1.4-mL sterile Micronic non-coded push cap U-bottom tubes and capped with a pierceable thermo plastic elastomer cap (MP32022; MP53007 [Nova Biostorage + (Canonsburg, PA, USA)]), snap frozen on dry ice, and stored frozen at approximately −80°C. Tumor samples were excised from the mouse and placed into 2 mL FastPrep tubes (Catalog No. 5076-400 [MP Biomedicals (Santa Ana, CA, USA)]), snap frozen on dry ice and stored frozen at approximately −80°C for pharmacokinetic (PK) analysis. Samples were shipped on dry ice to Charles River Laboratories (Worcester, MA, USA), where they were stored in a freezer set to maintain −60°C or colder until analysis.

The concentration of TAK-676 in the plasma and tumor samples was determined using a liquid chromatography with tandem mass spectrometry (LC–MS/MS) method (Charles River Laboratories; qualified method CW-0728-DB-AC-B09). The lower limit of quantitation (LLOQ) was 1.00 and 5.00 nM in mouse plasma and tumor homogenate, respectively. A protein precipitation extraction method was used for sample preparation. The LC–MS/MS setup was an MDS SCIEX API5500™ mass spectrometer (AB SCIEX [Concord, Ontario, Canada]) equipped with an Agilent 1260 LC System and LEAP autosampler. A reverse-phase gradient method running at a flow rate of 0.7 mL/minute on a Macmod ACE C18 AR column (2.1 mm ID × 50 mm; particle size 3 µm) was used for the analyte separation. The mobile phases used were water (A) and 50:50 MeCN:MeOH (B), and both were supplemented with formic acid (0.1%, volume-to-volume ratio [v:v]). TAK-676 was ionized under a positive ion spray mode and detected through the multiple-reaction monitoring (MRM) of a mass transition pair at a mass-to-charge ratio (m/z) of 711.0/330.0. Calibration curves of TAK-676 were established using standards, and the peak area ratios of the analyte against the internal standard (atenolol) were used to quantify samples. Using a plasma volume of 50 µL, linearity was achieved in the TAK-676 concentration range of 1 to 1000 nM. Using a tumor homogenate volume of 50 µL, linearity was achieved in the TAK-676 concentration range of 5 to 5000 nM.

PK analysis of plasma and tumor concentration data (rounded to 3 significant figures) was performed using Watson LIMS™ software, Version 7.5 (Thermo Scientific™ [Waltham, MA, USA]). Kinetic parameters were estimated using a noncompartmental model. Area under the concentration–time curve from 0 to 72 hours (AUC_72_) was calculated using the linear trapezoidal rule. Concentrations of TAK-676 that were below the quantitation limit were set to 0.00 ng/mL for the purpose of calculating mean concentrations and the PK parameters.

No statistical analyses were performed on the plasma and tumor concentration data or derived PK parameters. All derived parameters and associated standard deviations are reported to 3 significant figures, except for time to reach maximum observed concentration (C_max_) (t_max_,) which is reported to 2 significant figures. The conversion factor is 1 ng/mL = 1.41 nM based on molecular weight of 710.52. All doses, plasma and tumor concentrations, and derived PK parameters are presented as the free base.

***In vivo* antitumor activity in syngeneic CT26.WT, A20, and B16-F10 mouse models**

***CT26.WT model***

Nine-week-old female BALB/c mice were inoculated subcutaneously (SC) with 0.2 × 10^6^ CT26.WT tumor cells in the right flank. Tumor growth was monitored twice per week using vernier calipers and tumor volume was calculated using the formula (0.5 × [length × width^2^]). When the mean tumor volume (MTV) reached approximately 125 mm^3^, animals were randomized into treatment groups (*n* = 8/group). The individual groups were dosed intravenously (IV) with either vehicle (PBS) or TAK‑676 (1.0 or 2.0 mg/kg) on Days 0, 3, and 6.

Tumor size and body weight were measured twice per week. Tumor volume and body weight measurements for the initial efficacy portion of this study were recorded up to Day 66, with data up to Day 13 presented. Inhibition of tumor growth was determined by calculating the percent growth rate inhibition (GRI) on Day 13, which is when the vehicle group was removed from study because MTV reached approximately 3000 mm^3^. The differences in tumor growth trends over time between pairs of treatment groups and vehicle were assessed by fitting each animal’s data to a simple exponential growth model and comparing the mean growth rates of the 2 groups by Day 13. Treatment started on Day 0.

Complete regression (CR) was also used as an endpoint to evaluate efficacy. CR was defined as a decrease in tumor volume to an undetectable size (<25 mm^3^) for at least 30 days following the final dose of TAK-676.

*A20 model*

Nine-week-old female BALB/c mice were inoculated SC with 1.0 × 10^6^ A20 tumor cells in the right flank. Tumor growth and body weights were monitored on Days 0, 3, 6, 9, 13, 17, 28, 31, 34, 37, 41, 44, and 48 using vernier calipers and MTV was calculated using the formula: 0.5 × (length × width^2^). When the MTV reached approximately 100 mm^3^, animals were randomized into treatment groups (*n* = 10/group) and dosed IV with either vehicle (PBS), or TAK-676 (1 or 2 mg/kg) on Days 0, 3, and 6.

Tumor size and body weight were measured twice weekly. Tumor GRI was calculated on Day 9, which was the final day on which all 10 vehicle group animals remained on study. Tumor size and body weight measurements continued until the mice reached humane endpoints or until the study was terminated on Day 48.

Mean maximum body weight loss (BWL) was determined for each group using the mean body weight data from the treatment period, and the mean maximum percent body weight change was calculated on the basis of pre-dose body weights. Differences in tumor growth trends over time between pairs of treatment groups and vehicle were assessed by fitting each animal’s data to a simple exponential growth model and comparing the mean growth rates of the 2 groups by Day 9 when 6/10 control animals were removed from the study because their tumors had reached humane endpoints. The difference in the growth rates was summarized by the GRI.

*B16-F10 model*

Approximately 9-week-old female C57BL/6 were inoculated SC along the right flank with 0.08 × 10^6^ B16-F10 tumor cells. Tumor growth was monitored twice per week using calipers and MTV was calculated using the formula: 0.5 × (length × width^2^). When the MTV reached approximately 65 mm^3^, animals were randomized into 1 of 2 groups (*n* = 9/group) and dosed IV with either vehicle (PBS) or TAK 676 (2 mg/kg) on Days 0, 3, and 6.

Tumor size and body weight were measured biweekly until the MTV for a group reached approximately 1000 mm^3^ or until Day 20 (data presented up to Day 10). Treatment started on Day 0. GRI was calculated on Day 10 as that was the last measurement day that a statistically significant number of vehicle-treated mice remained for comparison. The treatment group was not terminated on this day, but individual growth graphs for every animal and mean BWL was shown up to Day 10. The animals remaining in the groups were monitored for as long as they remained on study up to Day 20 (data presented up to Day 10).

The mean maximum BWL was determined for each group using the mean body weight data from the treatment period, and the mean maximum percent body weight change was calculated on the basis of pre-dose body weights.

*B16-F10:* *C57BL/6J Tmem173gt/J (Goldenticket) model*

Approximately 12-week-old, female, C57BL/6J-Tmem173gt/J mice (Goldenticket) were inoculated SC along the right flank with 0.08 × 10^6^ B16-F10 tumor cells. Tumor growth was monitored twice per week using calipers and MTV was calculated using the formula: 0.5 × (length × width^2^). When the MTV reached approximately 66 mm^3^, animals were randomized into 2 treatment groups (*n* = 9/group) and dosed IV with either vehicle (PBS) or TAK‑676 (2 mg/kg) on Days 0, 3, and 6.

Tumor size and body weight were measured biweekly until the MTV for a group reached approximately 1000 mm^3^ or until Day 20 (data presented up to Day 10). Treatment started on Day 0. GRI was calculated on Day 10 as that was the last measurement day that a statistically significant number of vehicle-treated mice remained for comparison.

*Statistical analysis*

The differences in the tumor growth trends over time between pairs of treatment groups were assessed by fitting each animal’s data to a simple exponential growth model and comparing the mean growth rates of the 2 groups. The difference in the growth rates was summarized by the GRI, which is the reduction in growth rate experienced by the treatment group relative to that of the reference group, expressed as a fraction of the vehicle growth rate. A positive GRI indicates that the tumors in the treatment group grew at a reduced rate relative to the reference group. A statistically significant *P*-value (< 0.05) suggests that the trends over time for the 2 treatment groups were different. The growth of the syngeneic tumors used in these studies exhibit high growth rate; therefore, animals with a tumor volume greater than 10% of their body weight were removed from the study.

***In vivo* pharmacodynamic effects**

*Cytokine induction*

Six- to 8-week-old, female, BALB/c mice were inoculated SC with 1.0 × 10^6^ A20 tumor cells (cell suspension in RPMI 1640) in the right flank. A20 cells were acquired from American Type Culture Collection (ATCC), cultured, and stocked at Millennium according to the supplier’s recommendations. When tumors grew to an estimated 300 to 800 mm^3^, animals were assigned into groups (*n* = 5/timepoint). Each group of animals received a single dose of vehicle or TAK-676 (0.05, 0.125, 0.5, 1.0, or 2.0 mg/kg) by IV injection. Doses were calculated based on a 23 g body weight. Animals were sacrificed in accordance with IACUC guidelines at defined timepoints (3 and 6 hours post dose), and tumor and plasma were harvested according to approved ROPs.

For plasma collection, approximately 500 µL of whole blood was obtained via cardiac puncture, placed into tubes coated with dipotassium ethylenediaminetetraacetic acid (K_2_EDTA [Eppendorf Catalog No. 022379224]) to prevent clotting and centrifuged at 10,000 rpm for 5 minutes in a Heraeus Biofuge Fisco (Hanau, Germany) set at 4ºC. Approximately 200 µL of plasma was then transferred into U-bottom tubes and capped with a pierceable thermo plastic elastomer cap, snap frozen on dry ice, and stored frozen at approximately −80°C. Tumor samples were excised from the mouse and were placed into Covaris bags, snap frozen on dry ice and stored frozen at approximately −80°C.

Frozen tumor samples were dry pulverized using the Cryoprep Pulverizer according to manufacturer instructions and were sonicated and homogenized in 0.5% CHAPS (3-[(3-cholamidopropyl)dimethylammonio]-1-propanesulfonate) supplemented with protease and phosphatase inhibitors using the Focused-ultrasonicator as described in ROP DB_CP_015.01. The homogenized tumor samples were then transferred to appropriately labeled 1.5 mL micro-centrifuged tubes and centrifuged at 10,000 rpm in an Eppendorf Centrifuge 5417R, set at 4ºC for 10 minutes. The supernatant was then transferred to a new set of labeled 1.5 mL size micro-centrifuge tubes. The protein concentration of samples was determined using a Pierce BCA Protein Assay Kit.

For the ProcartaPlex^®^ Multiplex Immunoassay, plasma and tumor samples were thawed on ice, and centrifuged for 15 minutes at 10,000 rpm in an Allegra^®^ X-14R centrifuge set at 4ºC to rid of any debris in the samples. Supernatant was then collected for further processing.

Plasma and tumor supernatant samples were diluted 4 × for this assay to evaluate interferon alpha (IFN-α), interferon gamma (IFN-γ), monocyte chemoattractant protein-1 (MCP-1), tumor necrosis factor alpha (TNF-α), and interleukin-6 (IL-6). Plasma and tumor supernatant samples were diluted 16 × to evaluate the level of IFN-γ-induced protein 10 (IP-10). The concentrations of the 6 cytokines were measured using the Custom ProcartaPlex 6 Plex, according to the user manual. The assay was read using the Bio-Plex™ 200 System, with Bio-Plex Manager software, Version 6.1.1 (Bio-Rad Laboratories, Inc.).

Standard curves for individual cytokines were generated using 5-parameter logistic equation and the concentration of individual cytokines in each sample was calculated using Bio-Plex Manager software, Version 6.1.1. Samples with a concentration value below the LLOQ or above the upper limit of quantitation (ULOQ) were reported as having a value of LLOQ or ULOQ, respectively. Data (observed concentrations) were graphed using the GraphPad Prism software, Version 7.03. Individual values for each sample were graphed in a scatter plot format with the mean, standard deviation, and p value (where significant; relative to control) shown for each sample group.

*In vivo immune cell activation and proliferation*

Eight- to 10-week-old, female, C57/BL6 mice were inoculated SC with 0.08 × 10^6^ B16-F10 tumor cells (cell suspension in DMEM [Dulbecco’s Modified Eagle Medium]) in the right flank. When tumors grew to approximately 300 to 500 mm^3^, animals were randomly assigned into groups (*n* = 4/timepoint). Each group of animals received IV injections of either vehicle or TAK-676 (0.3, 1.0, or 2.0 mg/kg) on Days 0, 3, and 6. All mice with a tumor size above approximately 1000 mm^3^ were excluded. Animals were sacrificed at defined timepoints and tissue was harvested according to approved ROPs.

Tumor draining lymph nodes (axillary, brachial, and inguinal nodes on same side as the tumor) and tumor tissue were collected from each study animal on Days 3, 7, and 10 after the first dose. Six mice in the Day 14 groups were removed when tumor growth was determined to have met the humane endpoint. Because of insufficient data, this timepoint is not presented.

After removal of the tumor, approximately 0.5 g of tumor tissue was stored in RPMI medium supplemented with 10% heat inactivated FBS on ice until ready for processing. Tumor tissue was transferred to Miltenyi dissociation tubes supplemented with tumor dissociation enzyme mix (Miltenyi Biotec) and then placed on an OctoMacs tissue dissociator (Miltenyi Biotec). Samples were dissociated using the 37ºC_m_TDK_1 program. Tumor cell suspensions were passed through 70 µm cell strainers and the cells were pelleted by centrifugation for 5 minutes at 300 RCF in an Allegra^®^ X-14R centrifuge (Beckman Coulter Inc. [Brea, CA, USA]) set at 4ºC. To lyse RBCs, 2 mL of ammonium-chloride-potassium (ACK) lysing buffer (Thermo Fisher Scientific) was added to each tube and incubated for 5 minutes at room temperature. After incubation, 10 mL of RPMI 1640 media supplemented with 10% heat-inactivated FBS was added to each conical tube, and cells were pelleted for 5 minutes at 300 RCF in an Allegra^®^ X-14R centrifuge (Beckman Coulter Inc. [Brea, CA, USA]) set at 4ºC. After removing the supernatant, the pellet was resuspended with 1 mL of RPMI 1640 media supplemented in 10% heat-inactivated FBS and subjected to cell counting. After cell counting, the cells were pelleted for 10 minutes at 300 RCF in an Allegra^®^ X-14R centrifuge (Beckman Coulter Inc.) set at 4ºC. Samples were resuspended in RPMI 1640 media supplemented with 10% heat-inactivated FBS, 0.05 mM 2‑mercaptoethanol (BME), and 100 U/mL Pen-Strep to obtain a final concentration of 2 × 10^7^cells per mL. 100 µL of the single-cell suspension containing a total of 2 × 10^6^ cells was transferred to a 96-well U bottom plate for immediate staining with the DC flow cytometry staining panel. Another 100 µL of the single cell suspension containing a total of 2 × 10^6^ cells was transferred to a non-tissue culture-treated, flat-bottom plate with lid (Corning Inc.) and subjected to anti-CD3 and –CD28 stimulation. Surface staining and intracellular staining with a T-cell activation flow cytometry staining panel were performed after stimulation.

Lymph nodes (axillary, brachial, and inguinal nodes on the same side as the tumor) were removed from animals and placed into RPMI 1640 media, supplemented with 10% heat-inactivated FBS, 0.05 mM BME, and 100 U/mL Pen-Strep, and stored on ice until processed. All lymph nodes from the same animal were then transferred into a 70 µm cell strainer placed on top of a 50 mL conical tube. A 3 mL syringe top was used to macerate the lymph nodes completely. An additional 10 mL of RPMI 1640 media supplemented with 10% heat-inactivated FBS was added to wash each of the strainers and to ensure all cells were filtered through. The cells were then pelleted for 10 minutes at 500 RCF in an Allegra^®^ X-14R centrifuge (Beckman Coulter Inc. [Brea, CA, USA]) set at 4ºC. After removing the supernatant, the pellet was resuspended in 1mL of RPMI 1640 media supplemented with 10% heat-inactivated FBS, 0.05 mM BME, and 100 U/mL Pen-Strep. and subjected to cell counting. After cell counting, the cells were pelleted for 10 minutes at 300 RCF in an Allegra^®^ X‑14R centrifuge (Beckman Coulter Inc.) set at 4°C. Samples were resuspended in RPMI 1640 media supplemented with 10% heat-inactivated FBS, 0.05 mM BME, and 100 U/mL Pen-Strep to obtain a final concentration of 2 × 10^7^ cells per mL. 100 µL of the single-cell suspension containing a total of 2 × 10^6^ cells was transferred to a 96-well U bottom plate for immediate staining with a DC flow cytometry staining panel. Another 100 µL of the single-cell suspension containing a total of 2 × 10^6^ cells was transferred to a non-tissue culture-treated, flat-bottom plate with lid (Corning Inc.) and subjected to anti-CD3 and –CD28 stimulation. Surface staining and intracellular staining with a T-cell activation flow cytometry staining panel were performed after stimulation.

For cell counting, 50 mL of each sample, plus 450 mL of RPMI 1640 media (supplemented with 10% heat-inactivated FBS), was added to a Vi-CELL™ Sample Cup (4 × 120, Beckman Coulter, 383721) to reach a 1:10 dilution. Vi-Cell XR (Beckman Coulter, 383198) was used to determine concentration (cells/mL).

For cell stimulation, CD3ε monoclonal antibody (Thermo Fisher Scientific) was diluted in PBS to a final concentration of 10 µg/mL, and 100 µL was added to each well of a 96-well non-tissue culture-treated, flat-bottom plate with lid (Corning Inc.). The plate was incubated overnight (approximately 16 hours) at approximately 4ºC and washed twice with PBS to remove any excess unbound antibody. CD28 monoclonal antibody (Thermo Fisher Scientific) was diluted in RPMI 1640 media supplemented with 10% heat-inactivated FBS, 0.05mM BME, and 100 U/mL Pen-Strep to a concentration of 4 µg/mL (2´ concentration). 100 µL of the CD28 solution was added to each well of the previously coated anti-CD3ε plate. 100 µL of the single-cell suspensions containing a total 2 × 10^6^ cells were then added to each well and mixed thoroughly for a final concentration of 2 µg/mL of CD28 and a total volume of 200 µL. Unstimulated control wells containing neither CD3ε nor CD28 were also prepared. The cells were incubated overnight (approximately 16 hours) at approximately 37^o^C with 5% CO_2_. The following day, the cells were centrifuged briefly, 100 µL of media were removed from each well and replaced with 100 µL of RPMI 1640 media supplemented with 10% heat-inactivated FBS, 0.05 mM BME, 100 U/mL Pen-Strep, and GolgiPlug™ (1 µL/mL, BD Biosciences), and mixed thoroughly. The cells were incubated for an additional 5 hours at approximately 37ºC with 5% CO_2_. Cells were then transferred to 96-well U‑bottom plates for flow cytometry staining using a T-cell activation panel.

For flow cytometry staining, the 96-well U-bottom plates with single-cell suspensions of all samples were centrifuged for 4 minutes at 500 RCF in an Allegra^®^ X-14R centrifuge (Beckman Coulter Inc.) set at 4ºC. The supernatant was removed and washed once with 1 × PBS.

Subsequently, the cells were re-suspended in a solution of LIVE/DEAD™ Fixable Dead Cell Stain (Invitrogen)at a 1:1000 dilution in 1 × PBS without calcium and magnesium. The cells were stained at approximately 4ºC for 15 minutes while protected from light. After being stained, the cells were washed once in 1 × PBS and centrifuged for 4 minutes at 500 RCF in an Allegra^®^ X-14R centrifuge (Beckman Coulter Inc.) set at 4ºC. The cells were washed again in stain buffer (BD Pharmingen™) and centrifuged for 4 minutes at 500 RCF in an Allegra^®^ X-14R centrifuge (Beckman Coulter Inc.) set at 4ºC. Cells in each well of the 96-well U-bottom plate were re-suspended in 50 mL of Fc blocking solution containing 20 mg/mL Fc Block (BioXcell) in 1 × PBS. The plates were incubated at approximately 4ºC for 10 minutes while protected from light. After blocking, 50 mL of 2× staining mixture with the antibodies targeting surface antigens were added to all samples. The cells were stained at 4ºC for 30 minutes while protected from light. Cells were then pelleted for 4 minutes at 500 RCF in an Allegra^®^ X‑14R centrifuge (Beckman Coulter Inc.) set at 4ºC and washed twice with stain buffer. Cells were stored in stain buffer at 4ºC while protected from light until they were analyzed or processed for intracellular staining.

Following surface staining, cells were re-suspended in 1 × Fixation/Permeabilization Working Solution from the eBioscience™ Foxp3/Transcription Factor Staining Buffer Set (Invitrogen [Cambridge, MA, USA]) and incubated on ice for 30 minutes while being protected from light. Cells were pelleted for 4 minutes at 500 RCF in an Allegra^®^ X-14R centrifuge (Beckman Coulter Inc.) set at 4ºC, and washed with 1 × Permeabilization Buffer from the eBioscience™ Foxp3/Transcription Factor Staining Buffer Set. The cells were stained with intracellular antibodies in 1 × Permeabilization Buffer at approximately 4ºC for 30 minutes, while protected from light. After staining, cells were pelleted for 4 minutes at 500 RCF in an Allegra^®^ X-14R centrifuge (Beckman Coulter Inc.) set at 4ºC, and washed once with 1 × Permeabilization Buffer followed by one wash with stain buffer. Cells were stored in stain buffer until ready to be analyzed. Data acquisition for each sample was performed on an LSRFortessa™ (Becton, Dickinson, and Company [Franklin Lakes, NJ, USA]).

UltraComp eBeads™ Compensation Control Beads (Invitrogen [Cambridge, MA, USA]) for flow cytometry compensation were also stained with antibodies. One drop of UltraComp eBeads™ was added to each required well of a 96-well U‑bottom plate (1 drop per 0.5 mL of antibody tested). A single drop of UltraComp eBeads™ was also added to a well without any antibody as an unstained control for flow cytometry. For compensation of LIVE/DEAD dye, the Arc™ Amine Reactive Compensation Bead Kit (Invitrogen [Cambridge, MA, USA]) was used. One drop of Arc™ Amine Reactive Beads was also added in the unstained beads control along with the UltraComp eBeads™. The compensation control beads were stained and washed in parallel and with the same procedure used as for the immune cells.

Flow cytometric data files were analyzed using FlowJo^®^ software, Version 10.1 (FlowJo, LLC [Ashland, OR, USA]). For the T-cell activation panel, data were gated on CD45.2-positive, Live/Dead negative (–) to look at changes within the immune-cell compartment. These cells are presented as % Live CD45+ cells. From this gate, total T cells (defined as live/dead dye negative/CD45+/CD3+) were further gated on CD8+ cells and reported as % CD8+ T cells. The CD8+ T cells were then separated into IFN-γ positive and negative; the positive gate is reported as % IFN-γ+ CD8 T cells. The CD8+ T cells were also separated into Ki67-positive and -negative, and the positive gate reported as % Ki67+ CD8 T cells. Using the DC panel, CD11c/MHCI double-positive cells were gated off live CD45+ cells (described above) and reported as % CD11c+MHCI+ cells. Percentages of immune cell populations of interest were exported from FlowJo^®^ to Microsoft Office Excel 2007 (Microsoft, Redmond, WA, USA). The mean, standard deviation, and p value (relative to control) for each group was calculated and graphed using GraphPad Prism 7 software (GraphPad Software, [San Diego, CA, USA]).

**SUPPLEMENTAL RESULTS**

**TAK-676 is tolerated in syngeneic mouse models**

In mice bearing CT26.WT tumor cells, treatment with TAK 676 2.0 mg/kg IV on Days 0, 3, and 6 resulted in an average body weight loss (BWL) of 11.8% on Day 6, after which the mice recovered (Supplementary Fig. 3A). For mice bearing A20 tumor cells, TAK-676 1 and 2 mg/kg was generally tolerated, with a mean maximum BWL during the study of 3.8% in the TAK-676 2 mg/kg treatment group on Day 6. One mouse in the 1 mg/kg group died on Day 3 from an unknown cause (Supplementary Fig. 3B). Animals bearing B16-F10 tumor cells treated with TAK-676 demonstrated the greatest mean maximum BWL of 11.7% on Day 4 and there was 0% BWL loss by Day 10 (Supplementary Fig. 2C)

BWL for Goldenticket mice, and animals implanted with STING KO B16F10 tumor cells are shown in Supplementary Figure 4. In these experiments, WT C57BL/6 mice implanted with WT B16F10 tumor cells showed a mean maximum BWL of 11.7% on Day 4 for the TAK-676 group. WT C57BL/6 mice implanted with STING KO B16F10 tumor cells showed a mean maximum BWL of 5.4% on Day 4 for the TAK-676 treated group. In contrast, Goldenticket mice implanted with either WT or STING KO B16F10 tumor cells showed reduced BWL, with a mean maximum BWL of 1% (Day 6) and 2.3% (Day 3), respectively.
