# Supplementary material for: TAK-676: A Novel Stimulator of Interferon Genes (STING) Agonist Promoting Durable IFN-dependent Antitumor Immunity in Preclinical Studies
Source: Cancer Res Commun. 2022 Jun 23;2(6):489–502. doi: 10.1158/2767-9764.CRC-21-0161 (PMC10010323; doi:10.1158/2767-9764.CRC-21-0161)
Supplement: Supplementary Figures S1-S5 — Supplementary figures: Supplementary Figure 1 shows cell viability of human monocyte-derived dendric cells and mouse bone marrow dendritic cells. Supplementary Figure 2 shows mean plasma and tumor concentration-time curves of TAK-676 in BALB/C mice (A20 syngeneic tumors). Supplementary Figure 3 shows percentage body weight change in BALB/C mice (CT26.WT and A20 syngeneic tumors). Supplementary Figure 4 shows percentage body weight change in STING-deficient Goldenticket mice (STING WT and B16F10 syngeneic tumors). Supplementary Figure 5 shows cytokine expression in A20 tumor-bearing mice (plasma and tumor). [file crc-21-0161-s02.docx]

**TITLE:** TAK-676: A Novel Stimulator of Interferon Genes (STING) Agonist Promoting Durable Interferon-Dependent Anti-Tumor Immunity in Preclinical Studies

**Authors:**

Elizabeth Carideo Cunniff^1^*, Yosuke Sato^1^*, Doanh Mai^1^*, Vicky A. Appleman^1^, Shinji Iwasaki^2^, Vihren Kolev^1^, Atsushi Matsuda^2^, Judy Shi^1^, Michiyo Mochizuki^2^, Masato Yoshikawa^2^, Jian Huang^1^, Luhua Shen^1^, Satyajeet Haridas^1^, Vaishali Shinde^1^, Chris Gemski^1^, Emily R. Roberts^1^, Omid Ghasemi^1†^, Hojjat Bazzazi^1‡^, Saurabh Menon^1^, Tary Traore^1§^, Pu Shi^1¶^, Tennille D. Thelen^1^**, Joseph Conlon^1††^, Adnan O. Abu-Yousif^1^, Christopher Arendt^1^, Michael H. Shaw^1^, and Masanori Okaniwa^1^

*Authors contributed equally to this manuscript

**Affiliations:**

^1^Takeda Development Center Americas, Inc. (TDCA), Lexington, MA, USA

^2^Takeda Pharmaceutical Company, Ltd., Fujisawa, Kanagawa, Japan

^†^Current affiliation: Invicro, LLC, Needham, MA, USA

^‡^Current affiliation: Cytomx Therapeutics, South San Francisco, CA, USA

^§^Current affiliation: TScan Therapeutics, Waltham, MA, USA

^¶^Current affiliation: BeiGene, Cambridge, MA, USA

**Current affiliation: Atara Biotherapeutics, Inc., South San Francisco, CA, USA

^††^Current affiliation: Pfizer, Inflammation and Immunology, Cambridge, MA, USA

**Corresponding authors:**

Michael H. Shaw

Takeda Development Center Americas, Inc. (TDCA), 95 Hayden Avenue, Lexington, MA 02421, USA

Email: [michael.shaw2@takeda.com](mailto:michael.shaw2@takeda.com)

Phone: +1 (617) 761-6834

Masanori Okaniwa

Takeda Development Center Americas, Inc. (TDCA), 95 Hayden Avenue, Lexington, MA 02421, USA

Email: [Masanori.Okaniwa2@takeda.com](mailto:Masanori.Okaniwa2@takeda.com)

Phone: +1 (617) 444-1596

**Authors’ Disclosures**

YS, ECC, DM, VAA, SI, VK, AM, JS, MM, MY, JH, LS, SH, VS, CG, ERR, HB, SM, TT, PS, JC, AOA-Y, CWA, MHS, MO disclose employment with Takeda. OG discloses previous employment with Takeda and current employment with Invicro, LLC. TDT discloses previous employment with Takeda and current employment with Atara Biotherapeutics, Inc.

**SUPPLEMENTAL FIGURES**

**Supplementary Figure 1.** Cell viability of **A,** human monocyte derived dendritic cells by TAK-676 at 24 hours from 5 donors, and **B,** of mouse bone marrow derived dendritic cells up to 72 hours from 2 donors.


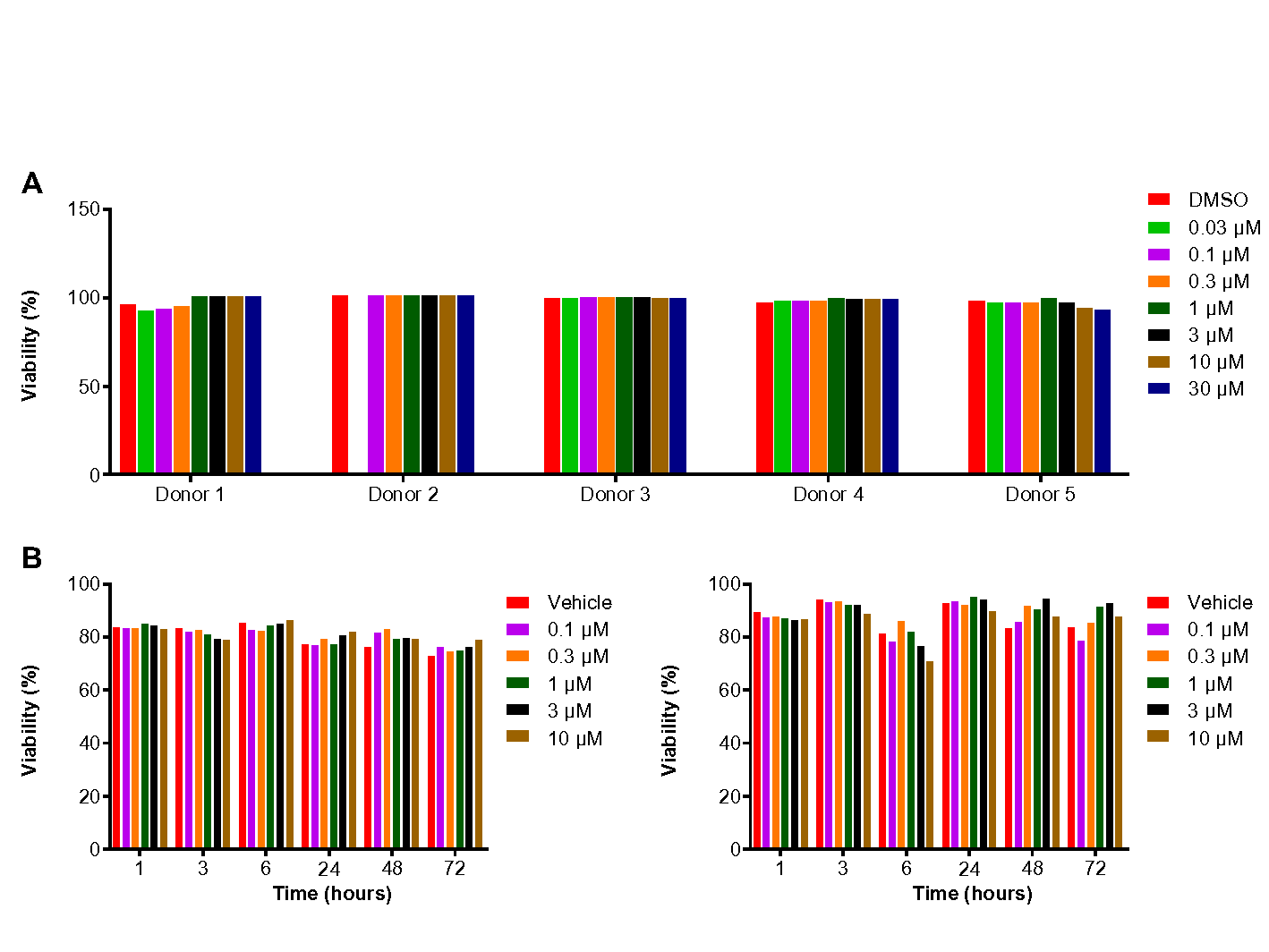


**Supplementary Figure 2.** Mean plasma (left) and tumor (right) concentration–time curves of TAK-676 in BALB/c mice bearing A20 tumors after intravenous administration of TAK-676 at 0.025, 0.125, 0.25, 0.5, and 2 mg/kg.


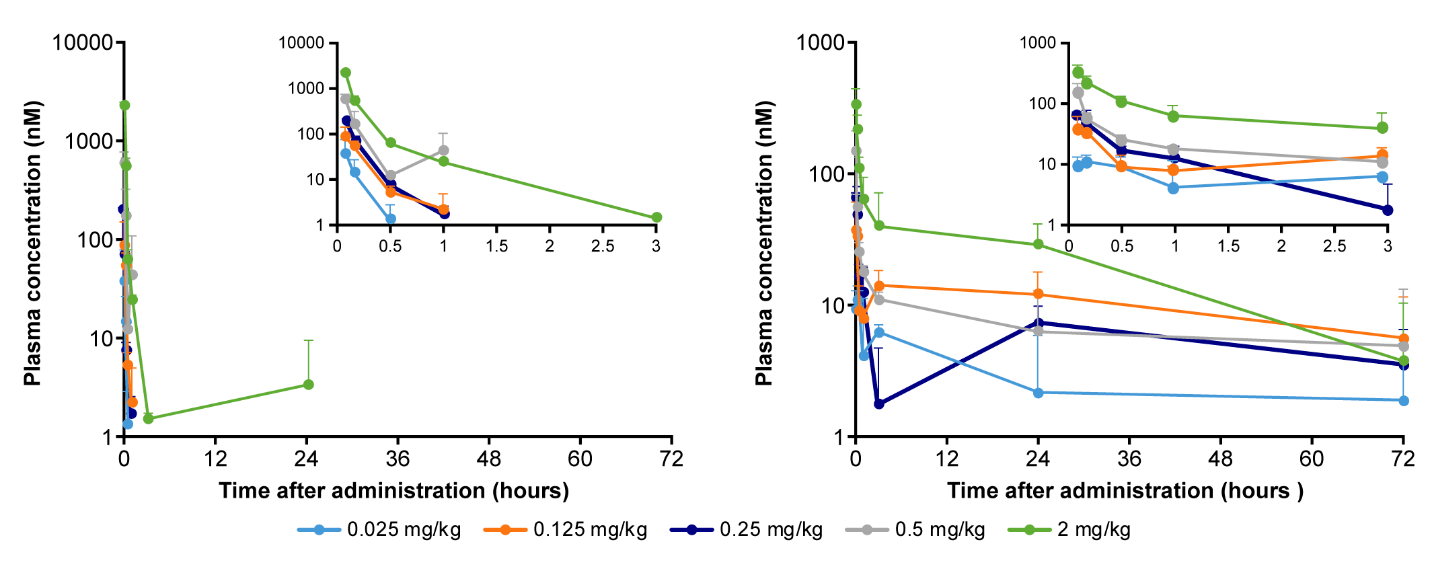


**Supplementary Figure 3.** Mean percent body weight change over time in: **A,** BALB/c mice bearing CT26.WT syngeneic tumors; **B,** BALB/c mice bearing A20 syngeneic tumors; All data shown are representative of at least three independent experiments.


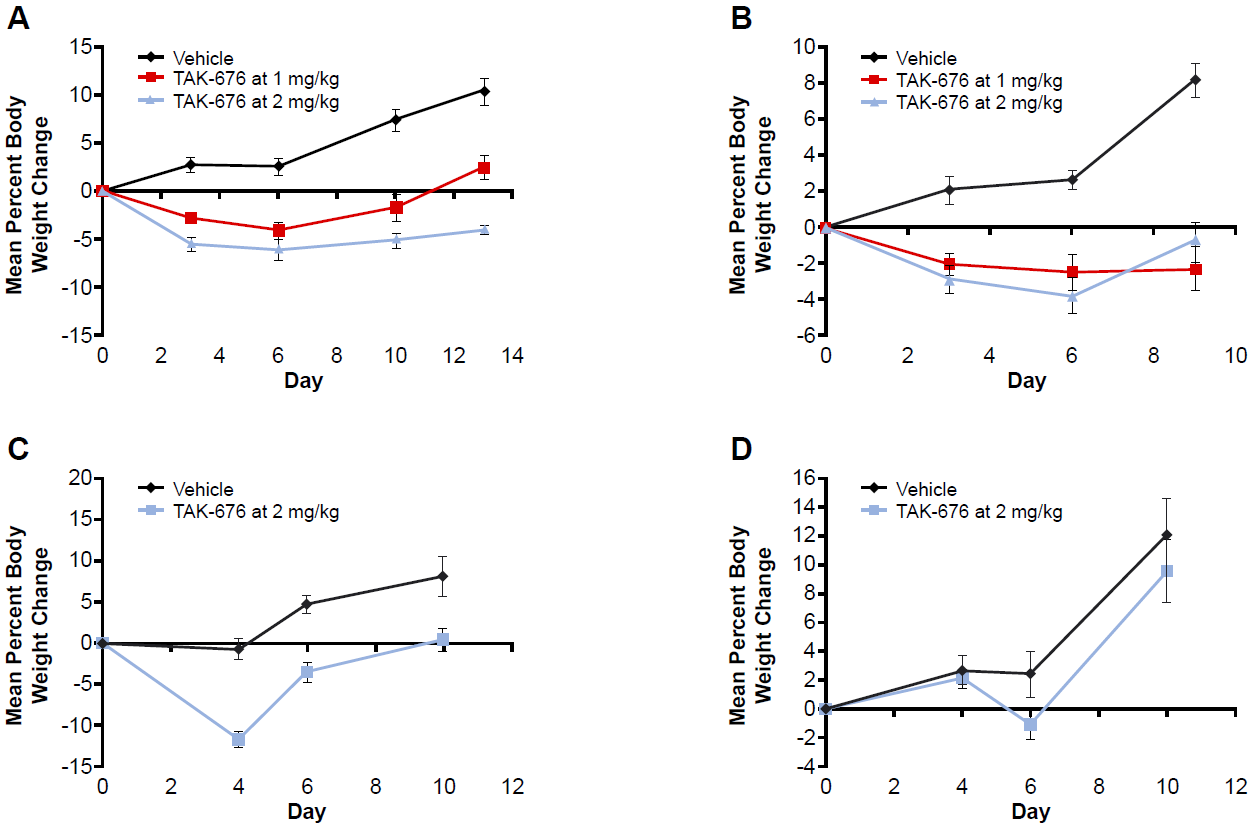


**Supplementary Figure 4.** Mean percent body weight change over time in WT or STING-deficient C57BL/6J-Tmem173gt/J (Goldenticket) mice bearing STING WT or deficient B16-F10 syngeneic tumors dosed with vehicle or TAK-676.


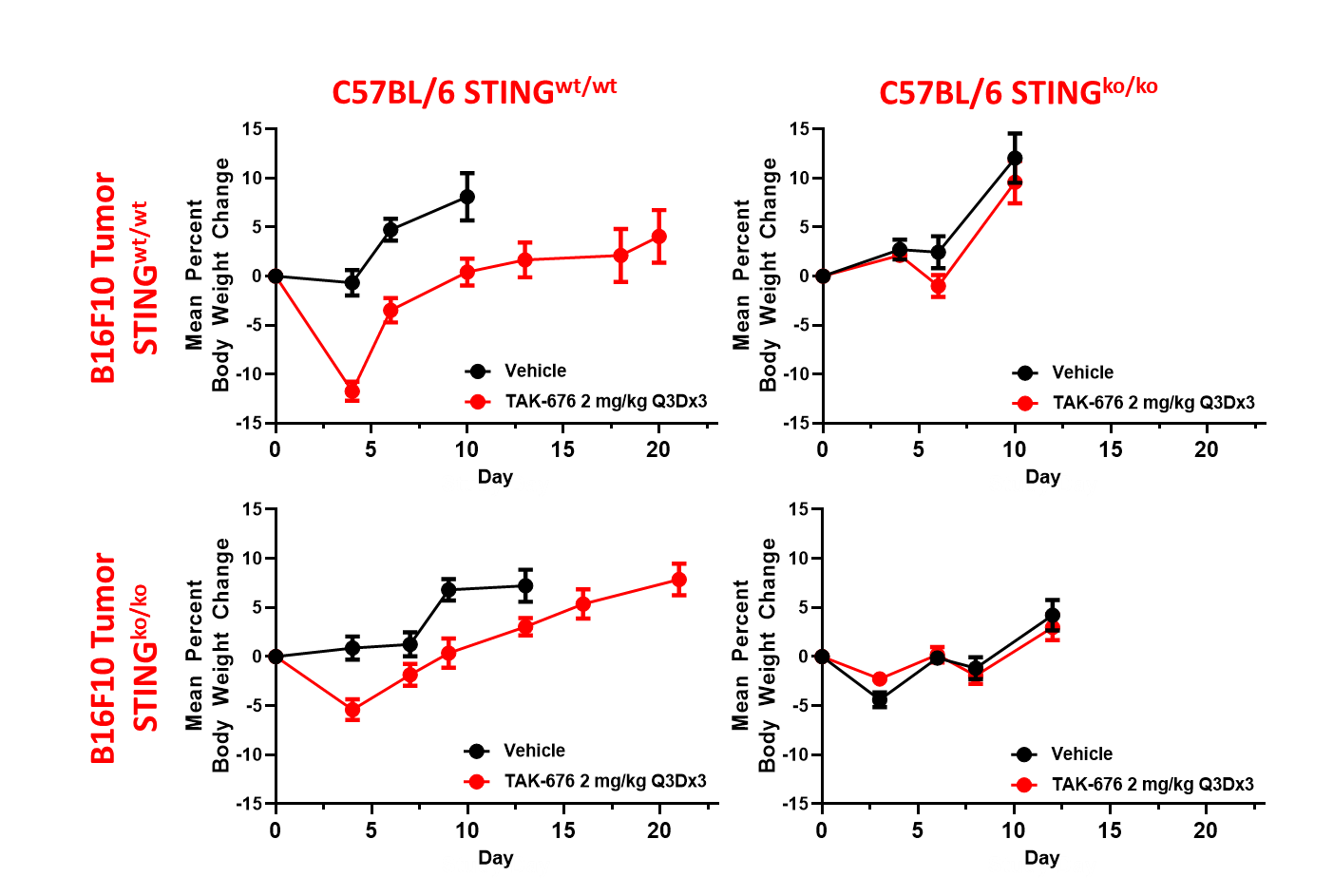


**Supplementary Figure 5**. Cytokine responses in plasma (left) and tumor (right) in A20 tumor-bearing mice following exposure to a single intravenous dose of vehicle or TAK-676: **A**, TNF-α; **B**, MCP-1; and **C**, IL-6. All data shown are representative of at least three independent experiments.


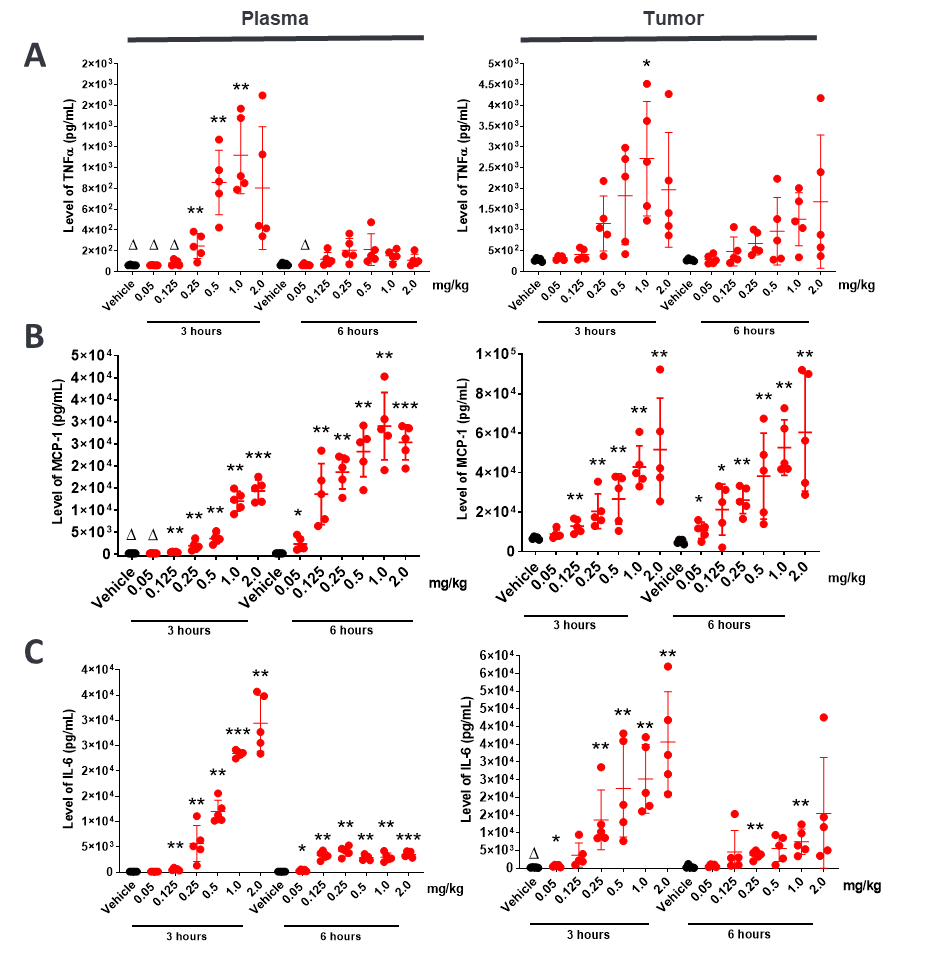
Δ: Indicates that some samples in this group had values below the lower limit of quantitation of the assay or had values extrapolated beyond the standard range.

*****: p value ≤ 0.05 relative to vehicle control.

**: p value ≤ 0.01 relative to vehicle control.

***: p value < 0.000001 relative to vehicle control.
