# Supplementary material for: TAK-676: A Novel Stimulator of Interferon Genes (STING) Agonist Promoting Durable IFN-dependent Antitumor Immunity in Preclinical Studies
Source: Cancer Res Commun. 2022 Jun 23;2(6):489–502. doi: 10.1158/2767-9764.CRC-21-0161 (PMC10010323; doi:10.1158/2767-9764.CRC-21-0161)
Supplement: Supplementary Tables S1-S3 — Supplementary tables: Supplementary Table 1 describes where cells were obtained from and details of authentication and mycoplasma testing dates. Supplementary Table 2 shows TAK-676 potency in cynomolgus monkey, human, mouse, and rat STING via time-resolved fluorescence resonance energy transfer assay. Supplementary Table 3 shows pharmacokinetic parameters (Cmax and AUC) after TAK-676 administration in BALB/C mice (A20 tumors). [file crc-21-0161-s03.docx]

**TITLE:** TAK-676: A Novel Stimulator of Interferon Genes (STING) Agonist Promoting Durable Interferon-Dependent Anti-Tumor Immunity in Preclinical Studies

**Authors:**

Elizabeth Carideo Cunniff^1^*, Yosuke Sato^1^*, Doanh Mai^1^*, Vicky A. Appleman^1^, Shinji Iwasaki^2^, Vihren Kolev^1^, Atsushi Matsuda^2^, Judy Shi^1^, Michiyo Mochizuki^2^, Masato Yoshikawa^2^, Jian Huang^1^, Luhua Shen^1^, Satyajeet Haridas^1^, Vaishali Shinde^1^, Chris Gemski^1^, Emily R. Roberts^1^, Omid Ghasemi^1†^, Hojjat Bazzazi^1^^‡^, Saurabh Menon^1^, Tary Traore^1^^§^, Pu Shi^1^^¶^, Tennille D. Thelen^1^**, Joseph Conlon^1^^††^, Adnan O. Abu-Yousif^1^, Christopher Arendt^1^, Michael H. Shaw^1^, and Masanori Okaniwa^1^

*Authors contributed equally to this manuscript

**Affiliations:**

^1^Takeda Development Center Americas, Inc. (TDCA), Lexington, MA, USA

^2^Takeda Pharmaceutical Company, Ltd., Fujisawa, Kanagawa, Japan

^†^Current affiliation: Invicro, LLC, Needham, MA, USA

^‡^Current affiliation: Cytomx Therapeutics, South San Francisco, CA, USA

^§^Current affiliation: TScan Therapeutics, Waltham, MA, USA

^¶^Current affiliation: BeiGene, Cambridge, MA, USA

**Current affiliation: Atara Biotherapeutics, Inc., South San Francisco, CA, USA

^††^Current affiliation: Pfizer, Inflammation and Immunology, Cambridge, MA, USA

**Corresponding authors:**

Michael H. Shaw

Takeda Development Center Americas, Inc. (TDCA), 95 Hayden Avenue, Lexington, MA 02421, USA

Email: [michael.shaw2@takeda.com](mailto:michael.shaw2@takeda.com)

Phone: +1 (617) 761-6834

Masanori Okaniwa

Takeda Development Center Americas, Inc. (TDCA), 95 Hayden Avenue, Lexington, MA 02421, USA

Email: [Masanori.Okaniwa2@takeda.com](mailto:Masanori.Okaniwa2@takeda.com)

Phone: +1 (617) 444-1596

**Authors’ Disclosures**

YS, ECC, DM, VAA, SI, VK, AM, JS, MM, MY, JH, LS, SH, VS, CG, ERR, HB, SM, TT, PS, JC, AOA-Y, CWA, MHS, MO disclose employment with Takeda. OG discloses previous employment with Takeda and current employment with Invicro, LLC. TDT discloses previous employment with Takeda and current employment with Atara Biotherapeutics, Inc.

**SUPPLEMENTAL TABLES**

**Supplementary Table 1. Cell line source, mycoplasma testing, and cell authentication**

| **Cell Line** | **Source** | **Catalog Number** | **Mycoplasma Testing Date** | **Authentication*** |
| --- | --- | --- | --- | --- |
| THP1-Dual™ Human Acute Myeloid Leukemia Cells | Invivogen | thpd-nfis | 6/19/2017 | No |
| Human Embryonic Kidney 293 (HEK293T) Cells | ATCC | CRL-11268 | 2/15/2018 | No |
| ISRE-Nano Luc HEK293T Cells | Promega | CS190901^†^ | 2/15/2018 | No |
| CT26.WT Cells | ATCC | CRL-2638 | 6/23/2016 | Yes |
| A20 Cells | ATCC | TIB-208 | 6/25/2015 | Yes |
| B16F10 Cells | ATCC | CRL-6475 | 7/22/2016 | Yes |

*Cells were authenticated by IDEXX BioAnalytics CellCheck cell line authentication service with
27-marker short tandem repeat strain analysis.

^†^Vector used at Promega to generate the cells.

**Supplementary Table 2.** Potency values of TAK-676 binding to mouse, rat, cynomolgus monkey, and human STING orthologs in TR-FRET-assay

| **Assay** | **Value** | **Units** | **Standard Deviation** | **n** |
| --- | --- | --- | --- | --- |
| mSTING TR-FRET IC_50_ | 0.010 | µM | 0.0008 | 3 |
| rSTING TR-FRET IC_50_ | 0.008 | µM | 0.001 | 3 |
| cSTING TR-FRET IC_50_ | 0.011 | µM | 0.001 | 3 |
| hSTING TR-FRET IC_50_ | 0.027 | µM | 0.008 | 20 |
| Values represent the arithmetic mean. All values were rounded. cSTING, cynomolgus monkey Stimulator of Interferon Genes; hSTING, human STING; IC_50_, concentration producing 50% inhibition; mSTING, mouse STING; rSTING, rat STING; SD, standard deviation; TRFRET, time-resolved fluorescence resonance energy transfer. | | | | |

**Supplementary Table 3.** Mean pharmacokinetic parameters in BALB/c mice bearing A20 tumors after intravenous administration of TAK-676 at 0.025, 0.125, 0.25, 0.5, and 2 mg/kg

| Matrix | Dose^a^ (mg/kg) | t_max_ (h) | C_max_ (nM) | C_max_/dose (nM)/(mg/kg) | AUC_72_ (nM*h) | AUC_72_/dose (nM*h)/(mg/kg) |
| --- | --- | --- | --- | --- | --- | --- |
| Plasma | 0.025 | 0.083 | 38.3 | 1530 | 11.0 | 438 |
|  | 0.125 | 0.083 | 86.6 | 693 | 29.4 | 235 |
|  | 0.25 | 0.083 | 255 | 1020 | 76.9 | 307 |
|  | 0.5 | 0.083 | 587 | 1170 | 227 | 455 |
|  | 2 | 0.083 | 2250 | 1130 | 872 | 436 |
| Tumor | 0.025 | 0.17 | 10.9 | 436 | 204 | 8170 |
|  | 0.125 | 0.083 | 37.9 | 303 | 742 | 5940 |
|  | 0.25 | 0.083 | 65.5 | 262 | 399 | 1600 |
|  | 0.5 | 0.083 | 151 | 302 | 522 | 1040 |
|  | 2 | 0.083 | 342 | 171 | 1770 | 883 |

^a^n=3 female BALB/c mice for each dose.

AUC_72_, area under the concentration-time curve from 0 to 72 hours; C_max_, maximum observed concentration; IV, intravenous; t_max_, time to reach C_max_ (in this case the plasma t_max_ was first sampling time after IV bolus).
